# Magnesium-catalyzed hydrosilylation of α,β-unsaturated esters†

**DOI:** 10.1039/c5sc02435h

**Published:** 2015-08-26

**Authors:** Nicole L. Lampland, Aradhana Pindwal, Steven R. Neal, Shealyn Schlauderaff, Arkady Ellern, Aaron D. Sadow

**Affiliations:** a Department of Chemistry, Iowa State University 1605 Gilman Hall Ames IA 50011 USA sadow@iastate.edu; b Department of Chemistry, University of Tennessee 515 Dabney-Buehler Hall, 1420 Circle Dr. Knoxville TN 37996 USA

## Abstract

To^M^MgHB(C_6_F_5_)_3_ (1, To^M^ = tris(4,4-dimethyl-2-oxazolinyl)phenylborate) catalyzes the 1,4-hydrosilylation of α,β-unsaturated esters. This magnesium hydridoborate compound is synthesized by the reaction of To^M^MgMe, PhSiH_3_, and B(C_6_F_5_)_3_. Unlike the transient To^M^MgH formed from the reaction of To^M^MgMe and PhSiH_3_, the borate adduct 1 persists in solution and in the solid state. Crystallographic characterization reveals tripodal coordination of the HB(C_6_F_5_)_3_ moiety to the six-coordinate magnesium center with a ∠Mg–H–B of 141(3)°. The pathway for formation of 1 is proposed to involve the reaction of To^M^MgMe and a PhSiH_3_/B(C_6_F_5_)_3_ adduct because the other possible intermediates, To^M^MgH and To^M^MgMeB(C_6_F_5_)_3_, react to give an intractable black solid and To^M^MgC_6_F_5_, respectively. Under catalytic conditions, silyl ketene acetals are isolated in high yield from the addition of hydrosilanes to α,β-unsaturated esters with 1 as the catalyst.

## Abstract 2

A magnesium hydridoborate compound catalyzes the 1,4-hydrosilylation of α,β-unsaturated esters to give silyl ketene acetals.

## Graphical abstract

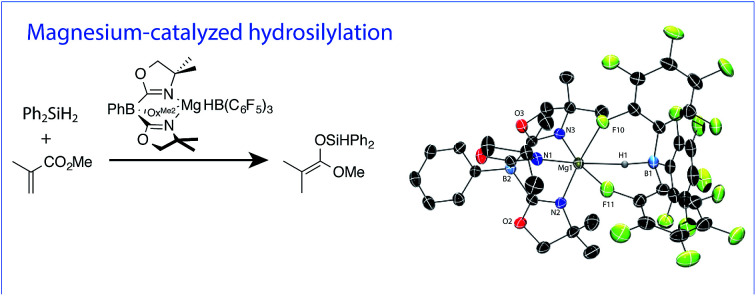

## Introduction

Catalytic addition reactions, such as hydrosilylation^1^ and hydroboration^2^ are important synthetic tools for the reduction of unsaturated moieties. These reactions also provide carbon-element, oxygen-element, and nitrogen-element bonds (element = silicon, boron, hydrogen) that allow further elaboration of organic and inorganic substances through cross-coupling^3^ or oxidation.^4^ Transition-metal, main-group metal, and rare earth metal complexes catalyze hydrosilylation through a range of pathways including 2-electron metal-centered redox chemistry, single-electron processes, σ-bond metathesis, or hydride abstraction reactions involving Lewis acid sites. Even a single compound can be involved in catalytic additions through a number of pathways that vary depending on the substrates, reductants, conditions and/or co-catalysts. For example, B(C_6_F_5_)_3_ catalyzes hydrosilylation of alkenes and carbonyls by action upon silanes,^5^ through frustrated Lewis Pairs in the presence of a bulky base,^6^ or through its combination with a metal center.^7^

The availability of many reaction pathways creates a challenge to control the selective conversion of carbonyl or olefin functional groups in substrates that contain both moieties. α,β-Unsaturated carbonyls can be particularly difficult because they may be susceptible to 1,2-addition to the carbonyl, 1,4-additions, α- or β-additions to the olefin, or polymerizations. The 1,4-addition products, silyl enol ethers or silyl ketene acetals, are valuable versatile nucleophiles in Mukaiyama aldol, Michael reactions,^8^ arylations,^9^ and haloketone or ketol formations. Since Wilkinson's and Karstedt's catalysts were shown to give selective 1,4-addition of R_3_SiH to α,β-unsaturated ketones,^10^ mainly platinum-group metals have been studied as catalysts for 1,4-hydrosilylation of α,β-unsaturated esters.^11^ Examples using more earth-abundant metals, such as main group or first row transition-metals, are less common and largely limited to Cu systems.^12^

There are only a few examples of alkene hydrosilylation catalyzed by heavy group 2 metal complexes (Ca, Sr, Ba),^13^ and carbonyl hydrosilylation is even less common. This is likely a result of the oxophilicity of magnesium and its heavier congeners. In fact, [{Me-Nacnac^Dipp^}CaH·THF]_2_ (Me-Nacnac^Dipp^ = ((2,6-iPr_2_C_6_H_3_)NCMe)_2_CH) provides a rare example of a group 2 catalyzed 1,2-hydrosilylation of ketones.^14^ In the stoichiometric dearomatization of pyridine and quinoline derivatives utilizing [{Me-Nacnac^Dipp^}Mg^*n*^Bu] and PhSiH_3_, it was found that PhSiH_3_ is insufficiently reactive to provide catalytic turnover.^15^ To the best of our knowledge, there are no previous reports of hydrosilylation catalyzed by homogeneous magnesium complexes.

More often, esters are cleaved under hydrosilylation conditions with first-row transition-metal catalysts,^16^ or with main group catalysts in hydroborations.^17^ In a magnesium catalyzed hydroboration of esters, the α,β-unsaturated ester reacts through C–O bond cleavage while the C=C bond is unaffected.^17*b*^ In that system, an important postulated intermediate, To^M^Mg{H(RO)Bpin} (To^M^ = tris(4,4-dimethyl-2-oxazolinyl)phenylborate; Bpin = boron pinacol ester), contains a boron–hydrogen bond. The [M]{H(RO)Bpin} motif contains features also associated with [M]{HB(C_6_F_5_)_3_} complexes,^18^ including oxygen or fluorine coordination to the metal center and a B–H → M interaction featuring a long M–H distance and nonlinear B–H–M angle. Recently, a {Me-Nacnac^Dipp^}MHB(C_6_F_5_)_3_ complex (M = Mg, Ca) was reported to catalyze the hydroboration of carbon dioxide,^19^ and this may suggest that hydroborates derived from B(C_6_F_5_)_3_ or HBpin may lead to new chemistry. Alternatively, a terminal magnesium hydride supported by a tetradentate monoanionic trimethylated tetraazacyclododecane ligand is stabilized by AliBu_3_, which coordinates to the amide moiety in the ancillary ligand rather than the nucleophilic hydride.^20^ The tris(oxazolinyl)borato magnesium catalyst precursors studied for hydroboration, namely To^M^MgMe or To^M^MgOR, do not mediate hydrosilylation of esters under the conditions tested, further suggesting that the boron center in To^M^Mg{H(RO)Bpin} provides a key feature for magnesium-catalyzed conversions of oxygenates.

The present study follows this idea to develop magnesium-catalyzed reductions of oxygenates employing organosilanes, rather than pinacolborane, as stoichiometric reductants. Here, we have incorporated the [M]HB(C_6_F_5_)_3_ motif into the complex To^M^MgHB(C_6_F_5_)_3_ (1) and report its reactivity as the first magnesium-catalyzed hydrosilylation. This transformation provides silyl ketene acetals through 1,4-hydrosilylation of α,β-unsaturated esters.

## Results and discussion

The monomeric magnesium methyl To^M^MgMe reacts slowly with organosilanes to provide Me–Si bond-containing compounds. For example, To^M^MgMe and PhSiH_3_ react in toluene-*d*_8_ to form PhMeSiH_2_ over 3 h at 100 °C (eqn (1)).1
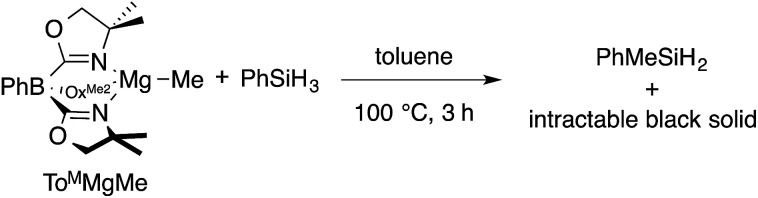


The presumed magnesium-containing product, To^M^MgH, is rapidly converted into an intractable black solid under these conditions. This black material is also formed as a byproduct in room temperature reactions of To^M^MgNHR and hydrosilanes that provide Si–N bond-containing products^21^ and in 1 : 1 reactions of To^M^MgMe and HBpin that afford Me-Bpin.^17*b*^ As a result, the identity of To^M^MgH is assumed based on reaction stoichiometry and its apparent reactivity as a catalytic intermediate.^21^ In order to obtain more evidence for To^M^MgH, we attempted to trap it as a Lewis acid adduct with B(C_6_F_5_)_3_.

A mixture of To^M^MgMe, PhSiH_3_, and B(C_6_F_5_)_3_ gives PhMeSiH_2_ and 1 (eqn (2)). Notably, this reaction occurs at room temperature over 10 min, whereas the direct interaction of To^M^MgMe and PhSiH_3_ requires the forcing conditions noted above. The optimized preparation of 1 involves dropwise addition of To^M^MgMe to a mixture of PhSiH_3_ and B(C_6_F_5_)_3_ dissolved in benzene.2
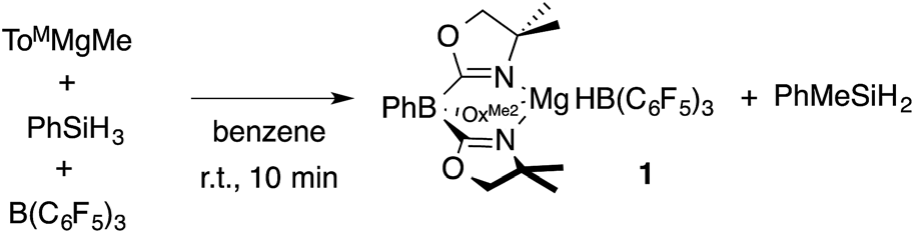


The ^1^H NMR spectrum of 1 (benzene-*d*_6_, r.t.) contained one set of oxazoline resonances, which is consistent with a pseudo-*C*_3v_-symmetric structure and tridentate coordination of To^M^ to the magnesium center. In addition, the hydrogen bonded to boron was observed at 2.7 ppm as a 1 : 1 : 1 : 1 quartet (^1^*J*_BH_ = 69 Hz). In the ^11^B NMR spectrum, a singlet at −18.2 ppm was assigned to the tris(oxazolinyl)borate ligand, and a doublet at −21.1 ppm (^1^*J*_HB_ = 69 Hz) characterized the HB(C_6_F_5_)_3_ group. The C_6_F_5_ are equivalent and freely rotating on the NMR timescale at room temperature, as indicated by the three resonances observed in the ^19^F NMR spectrum at −134.2, −156.5 and −161.4 ppm. The chemical shift of the *ortho*-F are similar to Cp*_2_ZrH{HB(C_6_F_5_)_3_} (Cp* = C_5_Me_5_), while the *meta*-F and *para*-F signals of 1 are downfield with respect to the zirconium hydride complex.^22^ The *δ*_*para*_−*δ*_*meta*_ of 5 ppm^23^ suggests coordination of HB(C_6_F_5_)_3_ to the Mg center. On the basis of these data and the single-crystal X-ray diffraction study (see below), ^19^F NMR spectra were acquired from 298 to 180 K; however, these signals did not vary over that temperature range. A single infrared band at 1579 cm^−1^ assigned to the oxazoline *ν*_CN_ also supported the assignment of tridentate To^M^-coordination. In addition, B–H bond formation was evidenced by an IR band at 2372 cm^−1^.

**Fig. 1:**
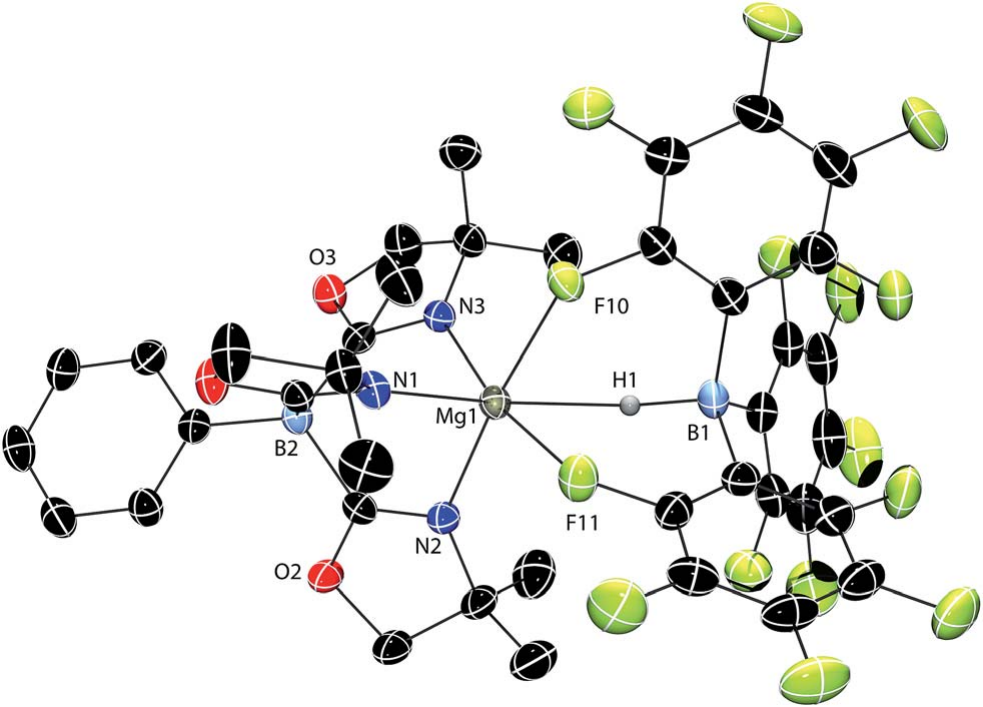
Rendered thermal ellipsoid diagram of To^M^MgHB(C_6_F_5_)_3_ (1) plotted at 50% probability. The H1, bridging between Mg1 and B1, was located objectively in the difference Fourier map and was refined isotropically. Two molecules of toluene and the H atoms on To^M^ are not included in the depiction for clarity.

A single crystal X-ray diffraction study confirms the identity of compound 1 as To^M^MgHB(C_6_F_5_)_3_, the tridentate coordination mode of the To^M^ ligand, and the tripodal Mg–HB(C_6_F_5_)_3_ interaction (Fig. 1). The six coordinating groups (three N from To^M^, two F and one H from HB(C_6_F_5_)_3_) form a distorted octahedral coordination geometry. Thus, the pseudo-*trans* disposed N1–Mg1–H1 angle is 162.3(7) and the N2–Mg1–F10 and N3–Mg1–F11 angles are 169.28(9) and 173.33(9)°. The Mg1–H1 and B1–H1 interatomic distances are 2.06(3) Å and 1.24(3) Å, respectively. The Mg1–H1 distance is longer than in the bridging Mg–H–Mg of [{Me-Nacnac^Dipp^}Mg(μ-H)]_2_ (1.95(3) Å)^24^ and [{*t*Bu-Nacnac^Dipp^}Mg(μ-H)]_2_ (1.80(5) and 1.91(5) Å; *t*Bu-Nacnac^Dipp^ = ((2,6-iPr_2_C_6_H_3_)NC*t*Bu)_2_CH). It is also longer than in the terminal magnesium hydride {*t*Bu-Nacnac^Dipp^}MgH(DMAP) (1.75(7) Å).^25^ The Mg1–H1 distance, however, is shorter than the related Mg–H distance of 2.19(3) Å in To^M^MgH_2_Bpin.^17*b*^ In [{Me-Nacnac^Dipp^}MgBH_4_]_2_, there are two types of Mg–H–B interactions, a Mg–H–B bridge (1.95(2) and 1.96(2) Å) containing shorter distances than in 1, and *Mg*,*Mg*,*B*-μ^3^-H with magnesium–hydrogen distances of 2.20(2) and 2.34(2) Å that are longer than 1.^26^ The B1–H1 distance of 1 is between the bridging (1.33(2) Å) and terminal (1.19(3) Å) B–H distances in diborane^27^ and much longer than in the terminal B–H (1.06(6) Å) of Cp*_2_ZrH{HB(C_6_F_5_)_3_}.^22^ Additionally, the B–H distance in 1 is similar to that of Cp*_2_SmHB(C_6_F_5_)_3_ (1.18(5) A),^18*b*^ Cp*_2_ScHB(C_6_F_5_)_3_ (1.14(3) Å),^18*a*^ and {Me-Nacnac^Dipp^}CaHB(C_6_F_5_)_3_ (1.16(2) Å).^19^

The nonlinear ∠Mg1–H1–B1 (141(3)°) angle is likely strongly influenced by the magnesium–fluorine interactions rather than from a Mg-(η^2^-H–B) interaction because the Mg1–B1 distance is long (3.149(4) Å). However, the Mg–H–B angle in To^M^MgH_2_Bpin of 93(2)° is much smaller, and as a result the Mg–B distance of 2.520(8) Å in the pinacol borane compound is shorter than in 1. The tridentate coordination mode of HB(C_6_F_5_)_3_ is similar in 1, MC(SiHMe_2_)_3_{HB(C_6_F_5_)_3_}THF_2_ (M = Ca, Yb),^18*d*^ and {Me-Nacnac^Dipp^}CaHB(C_6_F_5_)_3_.^19^ Cp*_2_SmHB(C_6_F_5_)_3_ contains two Sm−F interactions from the aryl rings and a possible interaction between Sm and the hydride.^18*b*^ Despite the size difference and the bulky tridentate oxazolinylborate ligand, Mg^2+^ still forms an analogous structure to these larger divalent metal cations. In contrast, Cp*_2_ScHB(C_6_F_5_)_3_ (ref. 18a) and Cp*_2_ZrH{HB(C_6_F_5_)_3_} (ref. 22) are bidentate through two M–F interactions.

**Scheme 1:**
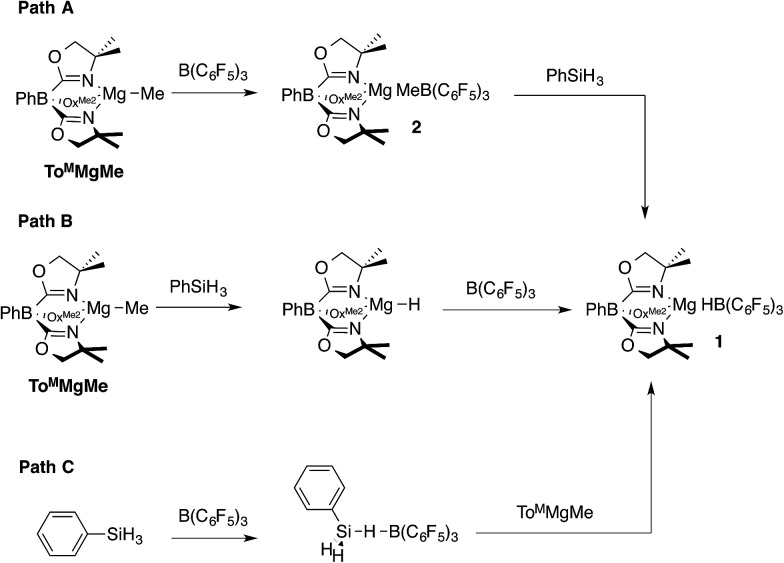
Possible pathways to To^M^MgHB(C_6_F_5_)_3_ (1).

Three pathways were considered for the formation of 1 (Scheme 1). The first one involves the reaction of To^M^MgMe and B(C_6_F_5_)_3_ to give To^M^MgMeB(C_6_F_5_)_3_ (2), followed by reaction of this species with PhSiH_3_ to give PhMeSiH_2_ and 1 (Path A). In Path B, the reaction of To^M^MgMe and PhSiH_3_ forms To^M^MgH, which is trapped by B(C_6_F_5_)_3_ to give 1. Alternatively, PhSiH_3_ and B(C_6_F_5_)_3_ could interact to give a transient adduct [PhH_2_SiHB(C_6_F_5_)_3_], and this intermediate reacts with To^M^MgMe to give the products (Path C). Methide abstraction by B(C_6_F_5_)_3_ in Path A is well established,^28^ supporting the possible intermediate To^M^MgMeB(C_6_F_5_)_3_. Furthermore, Cp*_2_ZrMe{(μ-Me)B(C_6_F_5_)_3_} is reported to undergo hydrogenation with H_2_ to give Cp*_2_ZrH{HB(C_6_F_5_)_3_},^22,28^ and (C_5_R_5_)_2_MMe{(μ-Me)B(C_6_F_5_)_3_} (M = Zr, Hf; C_5_R_5_ = C_5_H_5_, C_5_H_4_Me, C_5_Me_5_) and silanes react to give (C_5_R_5_)_2_MH{HB(C_6_F_5_)_3_}.^29^ These reactions, however, may involve methyl-hydride exchange through the conversion of [M]H{(μ-Me)B(C_6_F_5_)_3_} to [M]Me{(μ-H)B(C_6_F_5_)_3_} rather than direct hydrogenolysis of a M–Me–B bridge required for Path A. Path C is supported by proposed silane-borane adducts in B(C_6_F_5_)_3_-catalyzed hydrosilylations with tertiary silanes,^5*b*^ and recently a tris(pentafluorophenyl)-boraindene and triethylsilane adduct was isolated and fully characterized.^30^

Path B is immediately ruled out by the apparent reaction kinetics, which require forcing conditions to slowly generate To^M^MgH from PhSiH_3_ and To^M^MgMe. This reaction time and temperature contrasts the rapid formation of 1 from To^M^MgMe and PhSiH_3_ in the presence of B(C_6_F_5_)_3_. To test the feasibility of Path A, the proposed intermediate, To^M^MgMeB(C_6_F_5_)_3_ (2), was independently synthesized by addition of B(C_6_F_5_)_3_ dissolved in pentane to a benzene solution containing To^M^MgMe (eqn (3)).3
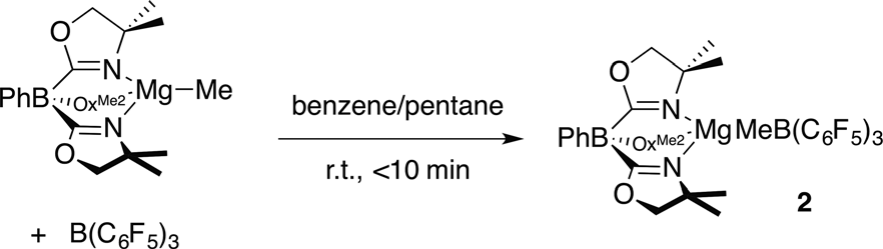


The product immediately precipitates giving analytically pure 2. Reactions in benzene-*d*_6_ or methylene chloride-*d*_2_ provide To^M^MgMeB(C_6_F_5_)_3_ as a partially soluble species that may be quickly characterized by solution-phase spectroscopy. However, once solvent is removed and To^M^MgMeB(C_6_F_5_)_3_ is isolated, it becomes insoluble in benzene and methylene chloride and only partially redissolves in THF. As in 1, ^1^H NMR spectra of *in situ* generated 2 revealed equivalent oxazoline groups. In an ^1^H–^11^B HMBC experiment, the resonance assigned to the MeB(C_6_F_5_)_3_ at 1.27 ppm correlated with a singlet ^11^B NMR signal at −15.5 ppm. However as 2 stands in benzene-*d*_6_, the signals for To^M^MgMeB(C_6_F_5_)_3_ decrease as the new species To^M^MgC_6_F_5_ (3) and BMe_3_ form. After 7 h, To^M^MgMeB(C_6_F_5_)_3_ is still the major component, but it is completely consumed over 20 h. This transformation occurs more rapidly in methylene chloride-*d*_2_ (*t*_1/2_ = 1 h).

Compound 3 is most conveniently prepared and isolated by the reaction of 1 equiv. of To^M^MgMe and 1 equiv. of B(C_6_F_5_)_3_ in benzene-*d*_6_ over 24 h, but also forms from the reaction of 0.3 equiv. of B(C_6_F_5_)_3_ with To^M^MgMe (eqn (4)). Solid To^M^MgC_6_F_5_ was purified from the BMe_3_ side product by washing with pentane.4
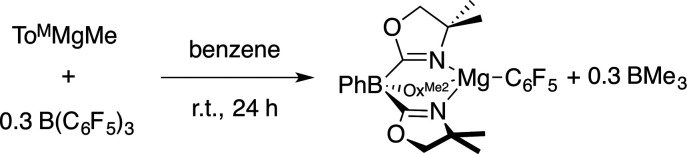


The ^1^H NMR spectrum of the crude reaction mixture contained a broad signal at 0.74 ppm assigned to BMe_3_ (ref. 31) and singlet resonances at 0.98 and 3.38 ppm assigned to the To^M^ ancillary ligand. Two peaks were observed in the ^11^B NMR spectrum at 86.5 and −18.3 ppm assigned to BMe_3_ and To^M^, respectively. In addition, the tridentate coordination of the tris(oxazolinyl)borate ligand is supported by the ^15^N NMR chemical shift of −158 ppm and the *ν*_CN_ band in the infrared spectrum at 1594 cm^−1^. These values are similar to those of crystallographically characterized To^M^MgMe (^15^N NMR: −157 ppm; *ν*_CN_: 1592 cm^−1^).^32^ Three signals in the ^19^F NMR spectrum included a downfield signal at −110 ppm assigned to the *ortho*-fluorine. For comparison, C_6_F_5_MgBr provides three sets of ^19^F NMR signals, with *ortho*-F resonance appearing 45 ppm upfield of the *para*-F peak.^33^

The reaction of *in situ* generated 2 and PhSiH_3_ at room temperature in benzene-*d*_6_ gives only starting materials after 30 min. Over *ca.* 24 h, To^M^MgMeB(C_6_F_5_)_3_ undergoes C_6_F_5_ transfer to the magnesium center, and PhSiH_3_ remains unconsumed. Micromolar-scale reactions in methylene chloride-*d*_2_ yield a mixture of To^M^MgC_6_F_5_, BMe_3_, B(C_6_F_5_)_3_, and PhSiH_3_ after 2 h. On the basis of these observations, 2 is not an intermediate in the formation of the magnesium hydridoborate 1, and Path A is ruled out. Therefore, the currently preferred pathway for the formation of 1 involves methide abstraction by a transient borane–silane adduct (Scheme 1, Path C). In fact, the aryl group transfer from boron to magnesium may be a decomposition pathway for 1 in catalytic reactions (see below).

α,β-Unsaturated esters and silanes react through selective 1,4-hydrosilylation in the presence of catalytic amounts of To^M^MgHB(C_6_F_5_)_3_ (1). For instance, the reaction of methyl methacrylate, Ph_2_SiH_2_, and 1 mol% 1 gives complete conversion of methyl methacrylate after 30 min in benzene-*d*_6_, as determined by ^1^H NMR spectroscopy (eqn (5)).5
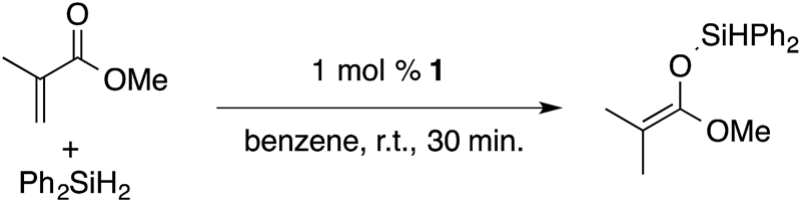


A ^1^H NMR spectrum of the isolated silyl ketene acetal product contained inequivalent methyl signals at 1.64 and 1.69 ppm, and singlets at 3.29 (3H) and 5.84 ppm (1H) assigned to the OMe and SiH groups. Olefinic signals, however, are not present in the product's ^1^H NMR spectrum. The ^13^C{^1^H} NMR spectrum contained a resonance at 150.93 ppm assigned to the acetal carbon. In an ^1^H–^29^Si HMBC experiment, a ^29^Si NMR signal at −14.5 ppm correlated to the SiH, inequivalent methyl signals, and phenyl resonances.

A range of silyl ketene acetals are prepared using 1 as the hydrosilylation catalyst (Table 1). Although transformations proceed with the low catalyst loadings of Table 1, scaled up reactions were performed with 20 mol% 1 to increase the rate of conversion. Secondary and tertiary silanes effectively hydrosilylate methyl methacrylate, and the products are isolated in good yield. In addition, the cyclic α,β-unsaturated ester 5,6-dihydro-2*H*-pyran-2-one react with PhMeSiH_2_ or BnMe_2_SiH in the presence of 1.

**Table 1:** 1-catalyzed hydrosilylation of α,β-unsaturated esters^a^

Reaction	mol% catalyst^b^	Time (h)	Isolated% yield
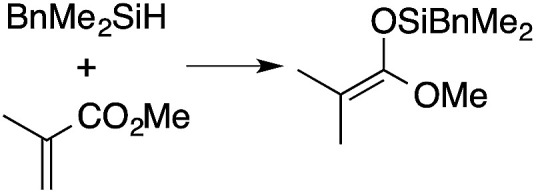	1	0.5	99
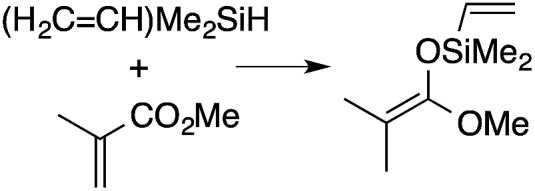	1	0.5	92
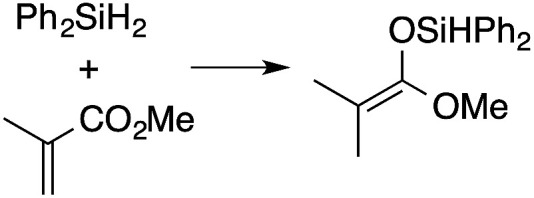	1	0.5	96
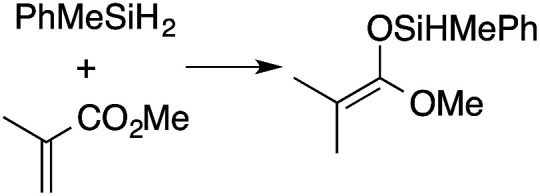	1	7^c^	97
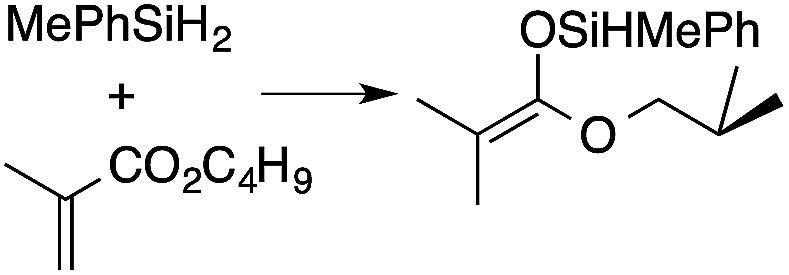	2.5	8	41
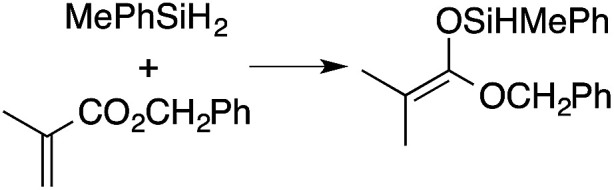	2.5	4	99
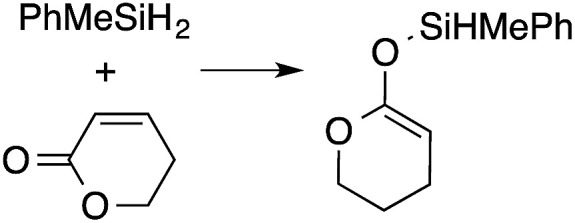	10	5	80
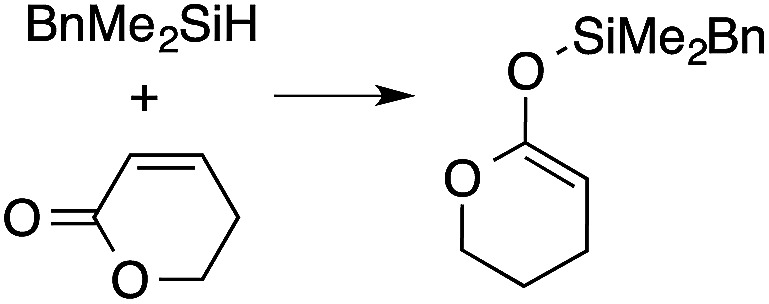	1	0.5^d^	83
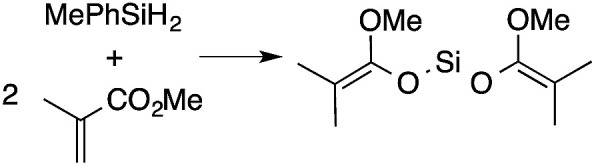	5	12^e^	32

a Reaction conditions: silane : acrylate = 1 : 1, benzene, r.t.

b *Catalyst loading given for NMR scale reactions.*

c 60 °C.

d 35 °C.

e 80 °C.

A number of experiments further test the key features of the catalyst structure and the reaction pathway. First, a series of To^M^MgX compounds (X = Me, C_6_F_5_, MeB(C_6_F_5_)_3_, B(C_6_F_5_)_4_) were investigated as catalysts for hydrosilylation of methyl methacrylate. A catalytic amount of To^M^MgMe reacts instantaneously with methyl methacrylate and PhMeSiH_2_ in benzene-*d*_6_ to give insoluble materials likely resulting from polymerization. Even though some of the silane is consumed in this reaction, neutral To^M^MgMe is not a viable hydrosilylation catalyst. Moreover, this further demonstrates that the silicon–oxygen bond formation is unlikely to involve σ-bond metathesis of silanes and a magnesium alkoxide.

In addition, ^1^H NMR spectra of catalytic mixtures of methyl methacrylate, PhMeSiH_2_ and 10 mol% To^M^MgMeB(C_6_F_5_)_3_ show only resonances assigned to methyl methacrylate and PhMeSiH_2_, and signals associated with the hydrosilylation product were not detected. To^M^MgMeB(C_6_F_5_)_3_ is converted to To^M^MgC_6_F_5_ under these conditions, and independent experiments show that To^M^MgC_6_F_5_ is also not catalytically active. Hydridoborate-free magnesium compounds were tested next. The reaction of To^M^MgMe and [Ph_3_C][B(C_6_F_5_)_4_] in benzene-*d*_6_ at room temperature gives [To^M^Mg][B(C_6_F_5_)_4_] as a precipitate after 15 min. However, this complex is not an ester hydrosilylation catalyst, and PhMeSiH_2_ and methyl methacrylate are unchanged after 2 d at 80 °C in the presence of 10 mol% [To^M^Mg][B(C_6_F_5_)_4_].

Alternatively, B(C_6_F_5_)_3_ is known as a hydrosilylation catalyst that mediates 1,2-addition of tertiary silanes to esters.^5*b*^ Free B(C_6_F_5_)_3_ might be present in the reaction mixture as a result of its dissociation from 1, so its catalytic mode of action in mixtures of silanes and α,β-unsaturated esters was probed. However upon treatment with 10 mol% B(C_6_F_5_)_3_, BnMe_2_SiH or (H_2_C=CH)Me_2_SiH and methacrylates provide mixtures containing the 1,4-addition product contaminated with at least 2 other species (see ESI† for spectra). The reactions of PhMeSiH_2_ and methyl methacylate, as catalyzed by 1 or 1 mol% B(C_6_F_5_)_3_, give inequivalent products. The product from the strong Lewis acid catalyst, in this case, does not contain an SiH, but is instead the double addition product PhMeSi{OC(OMe)=CMe_2_}_2_ formed as part of a mixture. The B(C_6_F_5_)_3_ catalyzed reaction of PhMeSiH_2_ and benzyl methacylate gives a complicated mixture. Interestingly, lower B(C_6_F_5_)_3_ loadings generally result in increased amounts of the side products with respect to silyl ketene acetal. These data indicate that the hydrosilylation of the methacrylates is not catalyzed by B(C_6_F_5_)_3_ when 1 is used as the catalyst. The B(C_6_F_5_)_3_-catalyzed reaction of Et_3_SiH and methyl methacrylate, however, gives the silyl ketene acetal quantitatively, as does the same conversion catalyzed by 1. Thus, B(C_6_F_5_)_3_-catalyzed hydrosilylations are more sensitive to the substitution of the organosilane than conversions catalyzed by 1.

Next, the interaction of 1 and organosilane was probed by ^1^H and ^11^B NMR spectroscopy. In the ^1^H NMR spectrum, the intensity of methyl and methylene signals associated with the oxazoline ligand in 1 diminish by *ca.* 70% upon addition of 10 equiv. of BnMe_2_SiH, and new, albeit small, oxazoline methyl and methylene signals were observed. The new oxazoline signals are not sufficiently abundant to account for all of the previous To^M^ signals. Moreover, the quartet at 2.7 ppm for HB(C_6_F_5_)_3_ was not visible after addition of excess organosilane, although a number of broad signals appeared in that region. The SiH of BnMe_2_SiH appeared as a sharp multiplet and was apparently unchanged in the presence of 1. The broad doublet at −21 ppm in the ^11^B NMR spectrum of 1 decreased in intensity, and a new signal at −24 ppm appeared. The new upfield ^11^B NMR signal appeared in the region typical of HB(C_6_F_5_)_3_, but H–B coupling was not resolved in the broad signal. At low temperature (190 K), the ^11^B NMR signal at −24 was not detected, and the doublet at −21 is the major HB(C_6_F_5_)_3_ resonance. As the temperature increased to 260 K, the broad signal at −24 ppm appeared while the doublet at −21 diminished. At the same time, the ^11^B NMR signal at −18 ppm for To^M^ was sharp at 190 K, broad at 260 K, and again sharpened at 280 K. These data suggest that BnMe_2_SiH and To^M^MgHB(C_6_F_5_)_3_ interact to disrupt the hydridoborate coordination to magnesium resulting in a dynamic system, but the HB(C_6_F_5_)_3_ moiety remains intact. Moreover, ^11^B NMR spectra acquired during catalytic conversions reveal signals at −18 and −24 ppm assigned to the boron centers in To^M^ and HB(C_6_F_5_)_3_. These two ^11^B NMR signals were also observed after complete conversion of methyl methacrylate *via* hydrosilylation. ^1^H NMR spectra of the catalytic reaction mixture, however, do not contain signals associated with 1. These data suggest that a fluxional derivative of 1 is involved in the catalytic conversion.

Under pseudo-first order conditions (using toluene-*d*_8_ as solvent) with excess methyl methacrylate, the half-life for the disappearance of Ph_2_SiH_2_ is ∼3 min at 64 °C, and over several minutes the silane is completely consumed. However, a methacrylate polymerization side-reaction interferes with kinetic measurements under these conditions. In the presence of excess Ph_2_SiH_2_ with respect to the methacrylate, zero-order, first-order, and second-order kinetic plots of methyl methacrylate concentration *vs.* time are non-linear, and complete conversion of the methacrylate is not obtained. The decrease in catalytic rate is even more prominent in methylene-chloride-*d*_2_ than in benzene-*d*_6_. In benzene-*d*_6_, the addition of methyl methacrylate and PhMeSiH_2_ is catalyzed by 10 mol% 1 in fewer than 10 min, while equivalent reaction conditions in methylene chloride-*d*_2_ give only 50% conversion after 24 h. Furthermore, the only To^M^-containing ^1^H NMR resonances observed in the catalytic reaction mixture (in methylene chloride-*d*_2_) were those assigned to To^M^MgC_6_F_5_. On the basis of faster conversion of To^M^MgMeB(C_6_F_5_)_3_ to To^M^MgC_6_F_5_ in methylene chloride than in benzene, the lack of activity of To^M^MgC_6_F_5_ as a hydrosilylation catalyst, and the lower catalytic activity in methylene chloride than in benzene, we suggest that catalyst deactivation occurs through C_6_F_5_ migration from boron to magnesium.

## Conclusions

The catalytic results above represent an unusual example of a magnesium-catalyzed hydrosilylation of C=O containing compounds. This catalytic transformation is particularly noteworthy in the context of the oxophilic magnesium center, and the general challenge of catalytic turnover under such reducing conditions. While a kinetically-characterized catalytic mechanism is not accessible in the current system, plausible intermediates can be considered, and some may be ruled out, on the basis of the observed reactivity of To^M^MgMe, To^M^MgHB(C_6_F_5_)_3_ (1), To^M^MgMeB(C_6_F_5_)_3_ (2), and To^M^MgC_6_F_5_ (3). The catalytic intermediates might involve the coordination of the ester oxygen to the magnesium center, a boron–carbon bond-containing species, a silane adduct of a cationic magnesium center, and/or an enolate of magnesium or boron. As one possibility, a magnesium enolate and a borane–silane adduct might interact to give Si–O bond formation and regenerate 1, following the proposed pathway for the formation of 1 from PhSiH_3_, To^M^MgMe, and B(C_6_F_5_)_3_. The catalysis requires [HB(C_6_F_5_)_3_]^−^, and no catalysis is observed with [B(C_6_F_5_)_4_]^−^ or with neutral magnesium alkyls To^M^MgMe or To^M^MgC_6_F_5_, providing additional support for the bifunctional role of 1 in this hydrosilylation, as proposed in frustrated Lewis pair chemistry.^34^ Moreover, To^M^MgMeB(C_6_F_5_)_3_ is not a viable hydrosilylation precatalyst, in contrast to To^M^MgHB(C_6_F_5_)_3_. This result further supports the postulate that the hydridoborate is key to accessing the active magnesium species.

A catalyst deactivation pathway is suggested to involve the transfer of C_6_F_5_ from boron to magnesium to give To^M^MgC_6_F_5_. To^M^MgC_6_F_5_ is shown to be catalytically inert and to form more rapidly in methylene chloride than in benzene; the trend of faster catalyst deactivation in methylene chloride than in benzene parallels the faster formation of To^M^MgC_6_F_5_ in the former solvent. These observations are taken as evidence in support of C_6_F_5_ transfer as a pathway to catalyst deactivation. This catalyst deactivation pathway is somewhat unexpected, given that magnesium alkyls are much more potent nucleophiles and bases than magnesium alkoxides. That is, in the presence of oxygen-containing substrates, a magnesium catalyst is deactivated by magnesium–carbon bond formation rather than magnesium–oxygen bond formation. This, and the catalytic hydrosilylation of oxygenates employing a highly oxophilic metal center, further indicates that the combination of a strong Lewis acid with early metal centers can access new reaction pathways through cooperation between the metal center and non-innocent counterion.

## Experimental

### To^M^MgHB(C_6_F_5_)_3_ (1)

A solution of To^M^MgMe (0.134 g, 0.32 mmol) dissolved in benzene was added in a dropwise fashion into a benzene solution containing PhSiH_3_ (0.069 g, 0.64 mmol) and B(C_6_F_5_)_3_ (0.162 g, 0.32 mmol). A white precipitate formed as the reaction mixture stirred for 30 min. The precipitate settled after centrifugation, and the supernatant was decanted. The white solid was washed with pentane (3 × 5 mL) and dried under vacuum, providing analytically pure To^M^MgHB(C_6_F_5_)_3_ (0.286 g, 0.31 mmol, 97.6%). Once isolated, To^M^MgHB(C_6_F_5_)_3_ is soluble in benzene or toluene, and X-ray quality single crystals were grown from a concentrated toluene solution at −30 °C. ^1^H NMR (600 MHz, benzene-*d*_6_): *δ* 0.82 (s, 18H, CNC*Me*_2_CH_2_O), 2.72 (br q, ^1^*J*_BH_ = 69 Hz, 1H, Mg*H*B(C_6_F_5_)_3_), 3.30 (s, 6H, CNCMe_2_C*H*_2_O), 7.38 (m, ^3^*J*_HH_ = 7.2 Hz, 1H, *para*-C_6_H_5_), 7.56 (m, ^3^*J*_HH_ = 7.6 Hz, 2H, *meta*-C_6_H_5_), 8.25 (d, ^3^*J*_HH_ = 7.2 Hz, 2H, *ortho*-C_6_H_5_). ^13^C{^1^H} NMR (150 MHz, THF-*d*_8_): *δ* 27.35 (CNC*Me*_2_CH_2_O), 66.13 (CN*C*Me_2_CH_2_O), 79.28 (CNCMe_2_*C*H_2_O), 130.24 (*para*-C_6_H_5_), 133.26 (*meta*-C_6_H_5_), 134.79 (C_6_F_5_), 135.74 (C_6_F_5_), 136.81 (C_6_F_5_), 137.49 (*ortho*-C_6_H_5_), 138.41 (C_6_F_5_), 142 (br, *ipso*-C_6_H_5_), 147.78 (C_6_F_5_), 149.35 (C_6_F_5_), 191 (*C*NCMe_2_CH_2_O). ^11^B NMR (192 MHz, benzene-*d*_6_): *δ* −18.2 (To^M^), −21.1 (d, ^1^*J*_HB_ = 69 Hz, MgHB(C_6_F_5_)_3_). ^19^F NMR (544 MHz, benzene-*d*_6_): *δ* −134.2 (*ortho*-C_6_F_5_), −156.5 (*para*-C_6_F_5_), −161.4 (*meta*-C_6_F_5_). ^15^N NMR (60 MHz, benzene-*d*_6_): *δ* −162. IR (KBr, cm^−1^): *ν* 2976 (s), 2937 (s), 2372 (w br, BH), 1642 (m), 1579 (s), 1511 (s), 1459 (s br), 1373 (m), 1271 (m), 1199 (m), 1180 (m), 1161 (m), 1087 (s), 965 (s br), 843 (w), 804 (w), 735 (w), 705 (w). Anal. calcd for C_39_H_30_B_2_F_15_MgN_3_O_3_: C, 50.94; H, 3.29; N, 4.57. Found C, 51.38; H, 3.41; N, 4.31. Mp: 166–167 °C.

### Crystallography

#### Crystal structure determination for compound 1

C_39_H_30_B_2_F_15_MgN_3_O_3_(C_7_H_8_)_2.5_, *M* = 1149.94, triclinic, *a* = 11.7916(18), *b* = 13.460(2), *c* = 18.239(3), *α* = 86.434(3), *β* = 88.133(3), *γ* = 69.802(3), *V* = 2711.3(7) Å^3^, *T* = 173 K, space group *P̄*1, *Z* = 2, 15 346 reflections measured, 9197 unique (*R*_int_ = 0.0303). The final *R*_1_(*F*^2^) and w*R*_2_(*F*^2^) for *I* > 2*σ*(*I*) were 0.0492 and 0.161.

### Representative catalytic hydrosilylation

#### Reaction of Ph_2_SiH_2_ and methyl methacrylate

To^M^MgHB(C_6_F_5_)_3_ (0.011 g, 0.012 mmol), methyl methacrylate (0.117 g, 1.17 mmol), and Ph_2_SiH_2_ (0.216 g, 1.17 mmol) were stirred in C_6_H_6_ for 30 min at room temperature. Benzene was removed under reduced pressure, leaving behind a colorless gel. The product was extracted with pentane, and the extracts were evaporated under reduced pressure to afford a colorless liquid (0.331 g, 1.13 mmol, 96.3%). ^1^H NMR (400 MHz, benzene-*d*_6_): *δ* 1.64 (s, 3H, C=C*Me*_2_), 1.69 (s, 3H, C=C*Me*_2_), 3.29 (s, 3H, OMe), 5.84 (s, 1H, SiH), 7.17 (m, 6H, C_6_H_5_), 7.74 (m, 4H, C_6_H_5_). ^13^C{^1^H} NMR (150 MHz, benzene-*d*_6_): *δ* 16.79 (C=C*Me*_2_), 17.42 (C=C*Me*_2_), 57.92 (OMe), 91.74 (C=*C*Me_2_), 130.47 (C_6_H_5_), 131.12 (C_6_H_5_), 134.16 (*ipso*-C_6_H_5_), 135.49 (C_6_H_5_), 136.39 (C_6_H_5_), 150.93 (*C*=CMe_2_). ^29^Si (119 MHz, benzene-*d*_6_) *δ* −14.5 (d, ^1^*J*_SiH_ = 201 Hz). IR (KBr, cm^−1^): *ν* 3094 (m), 2931 (s), 2158 (s), 1716 (s), 1661 (w), 1598 (m), 1566 (w), 1548 (w), 1528 (w), 1437 (s), 1263 (m), 1169 (br s), 1029 (m), 949 (m), 858 (s), 738 (s), 701 (s), 671 (w). Anal. calcd for C_17_H_20_O_2_Si: C, 71.79; H, 7.09. Found C, 71.61; H, 7.32.

## Supplementary Material

SC-006-C5SC02435H-s001

SC-006-C5SC02435H-s002

SC-006-C5SC02435H-s003
