# Supplementary material for: Magnesium-catalyzed hydrosilylation of α,β-unsaturated esters
Source: Chem Sci. 2015 Aug 26;6(12):6901–7. doi: 10.1039/c5sc02435h (PMC5947512; doi:10.1039/c5sc02435h)
Supplement: SC-006-C5SC02435H-s001 [file SC-006-C5SC02435H-s001.pdf]

Electronic Supplementary Material (ESI) for Chemical Science.
This journal is © The Royal Society of Chemistry 2015

# Electronic Supplementary Information for: Magnesium-catalyzed hydrosilylation of α,β-unsaturated esters

Nicole L. Lampland, Aradhana Pindwal, Steven R. Neal, Shealyn Schlauderaff, Arkady Ellern, and Aaron D. Sadow*

1605 Gilman Hall, Department of Chemistry, Iowa State University, Ames IA, 50011, USA

**General Procedures.** All reactions were performed under a dry argon atmosphere using standard Schlenk techniques or under a nitrogen atmosphere in a glovebox, unless otherwise indicated. Benzene, toluene, pentane, diethyl ether, and tetrahydrofuran were dried and deoxygenated using an IT PureSolv system. THF-$d_8$, methylene chloride-$d_2$, and benzene-$d_6$ were heated to reflux over Na/K alloy and vacuum-transferred. $To^MMgMe$,[1] $B(C_6F_5)_3$,[2] and $PhSiH_3$[3] were synthesized according to literature procedures. $PhMeSiH_2$ and $BnMe_2SiH$ were prepared by reaction of the chlorosilane with $LiAlH_4$. Vinyldimethylsilane, diphenylsilane, and triethylsilane were purchased from Gelest and purified by distillation under a dry argon atmosphere prior to use. Methyl methacrylate, benzyl methacrylate, and isobutyl methacrylate were purchased from Alfa Aesar and degassed by three freeze-pump-thaw cycles prior to use. 5,6-Dihydro-2*H*-pyran-2-one was purchased from Acros and degassed by three freeze-pump-thaw cycles prior to use. $^1H$, $^{13}C\{^1H\}$, and $^{11}B$ NMR spectra were collected on a Bruker AVII 600 spectrometer, a Bruker DRX-400 spectrometer, or a MR-400 spectrometer. $^{29}Si$ INEPT and $^1H$-$^{29}Si$ HMBC spectra were collected on a Bruker AVII 600 spectrometer. $^{15}N$ chemical shifts were determined by $^1H$-$^{15}N$ HMBC experiments on a Bruker AVII 600 spectrometer with a Bruker Z-gradient inverse TXI $^1H/^{13}C/^{15}N$ 5mm cryoprobe; $^{15}N$ chemical shifts were originally

referenced to an external liquid $NH_3$ standard and recalculated to the $CH_3NO_2$ chemical shift scale by adding -381.9 ppm. Elemental analyses were performed using a Perkin-Elmer 2400 Series II CHN/S by the Iowa State Chemical Instrumentation Facility. X-ray diffraction data was collected on a Bruker APEX II diffractometer.

**$To^M MgHB(C_6F_5)_3$ (1).** A solution of $To^M MgMe$ (0.134 g, 0.32 mmol) dissolved in benzene was added in a dropwise fashion into a benzene solution containing $PhSiH_3$ (0.069 g, 0.64 mmol) and $B(C_6F_5)_3$ (0.162 g, 0.32 mmol). A white precipitate formed as the reaction mixture stirred for 30 min. The precipitate settled after centrifugation, and the supernatant was decanted. The white solid was washed with pentane (3 × 5 mL) and dried under vacuum, providing analytically pure $To^M MgHB(C_6F_5)_3$ (0.286 g, 0.31 mmol, 97.6%). Once isolated, $To^M MgHB(C_6F_5)_3$ is soluble in benzene or toluene, and X-ray quality single crystals were grown from a concentrated toluene solution at –30 °C. $^1H$ NMR (600 MHz, benzene-$d_6$): δ 0.82 (s, 18 H, $CNC\mathit{Me}_2CH_2O$), 2.72 (br q, $^1J_{BH}$ = 68 Hz, 1 H, $Mg\mathit{H}B(C_6F_5)_3$), 3.30 (s, 6 H, $CNCMe_2C\mathit{H}_2O$), 7.38 (m, $^3J_{HH}$ = 7.2 Hz, 1 H, *para*-$C_6H_5$), 7.56 (m, $^3J_{HH}$ = 7.6 Hz, 2 H, *meta*-$C_6H_5$), 8.25 (d, $^3J_{HH}$ = 7.2 Hz, 2 H, *ortho*-$C_6H_5$). $^{13}C\{^1H\}$ NMR (150 MHz, THF-$d_8$): δ 27.35 ($CNC\mathit{Me}_2CH_2O$, overlapping with THF-$d_8$), 66.13 ($CN\mathit{C}Me_2CH_2O$, overlapping with THF-$d_8$), 79.28 ($CNCMe_2\mathit{C}H_2O$), 130.24 (*para*-$C_6H_5$), 133.26 (*meta*-$C_6H_5$), 134.79 ($C_6F_5$), 135.74 ($C_6F_5$), 136.81 ($C_6F_5$), 137.49 (*ortho*-$C_6H_5$), 138.41 ($C_6F_5$), 142 (br, *ipso*-$C_6H_5$), 147.78 ($C_6F_5$), 149.35 ($C_6F_5$), 191 ($\mathit{C}NCMe_2CH_2O$). $^{11}B$ NMR (192 MHz, benzene-$d_6$): δ –18.2 ($To^M$), –21.1 (d, $^1J_{HB}$ = 69 Hz, $MgHB(C_6F_5)_3$). $^{19}F$ NMR (544 MHz, benzene-$d_6$): δ –134.2 (*ortho*-$C_6F_5$), –156.5 (*para*-$C_6F_5$), –161.4 (*meta*-$C_6F_5$). $^{15}N$ NMR (60 MHz, benzene-$d_6$): δ –162. IR (KBr, $cm^{-1}$): ν 2976 (s), 2937 (s), 2372 (w br, BH), 1642 (m), 1579 (s), 1511 (s), 1459 (s br), 1373 (m), 1271 (m), 1199 (m), 1180 (m), 1161 (m), 1087 (s), 965

(s br), 843 (w), 804 (w), 735 (w), 705 (w). Anal. Calcd. for $C_{39}H_{30}B_2F_{15}MgN_3O_3$: C, 50.94; H, 3.29; N, 4.57. Found C, 51.38; H, 3.41; N, 4.31. Mp: 166-167 °C.

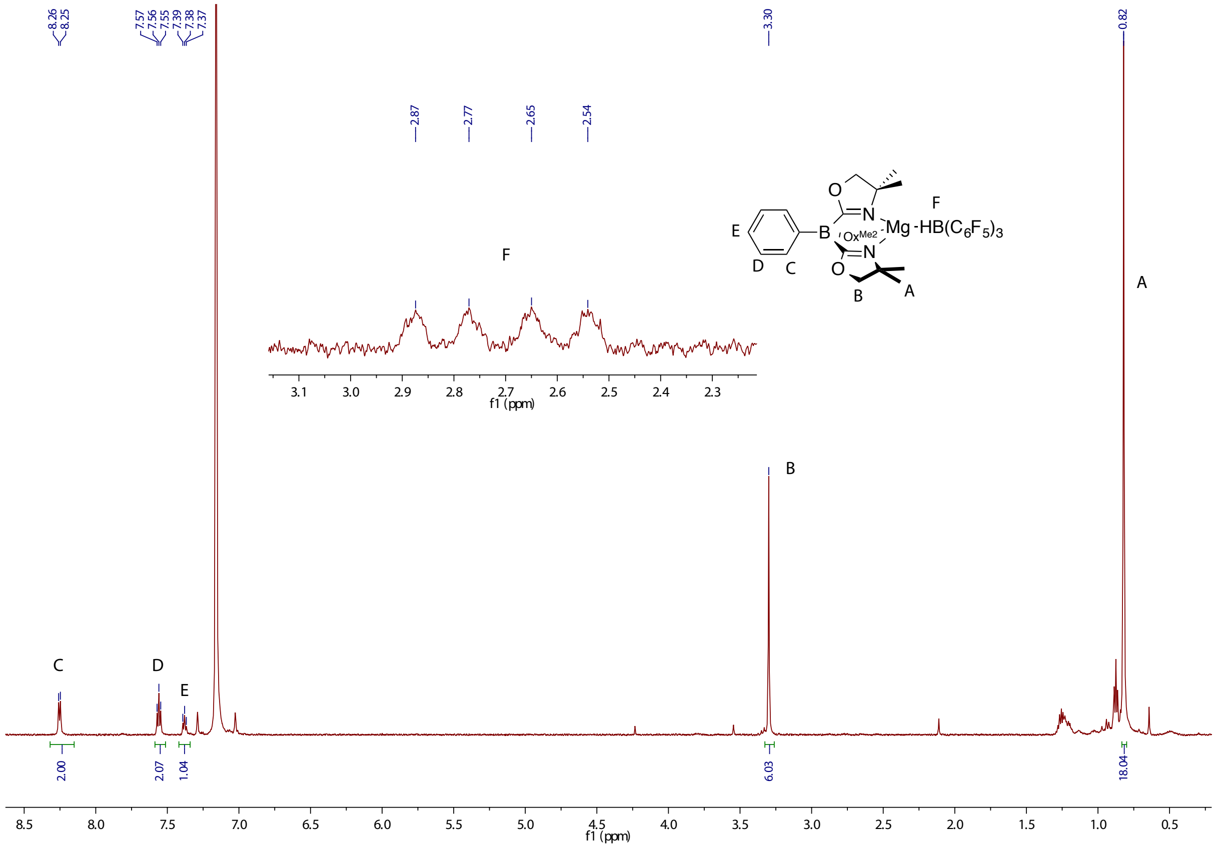

**Figure S1.** $^1$H NMR spectrum (600 MHz, benzene-$d_6$) of $To^MMgHB(C_6F_5)_3$.

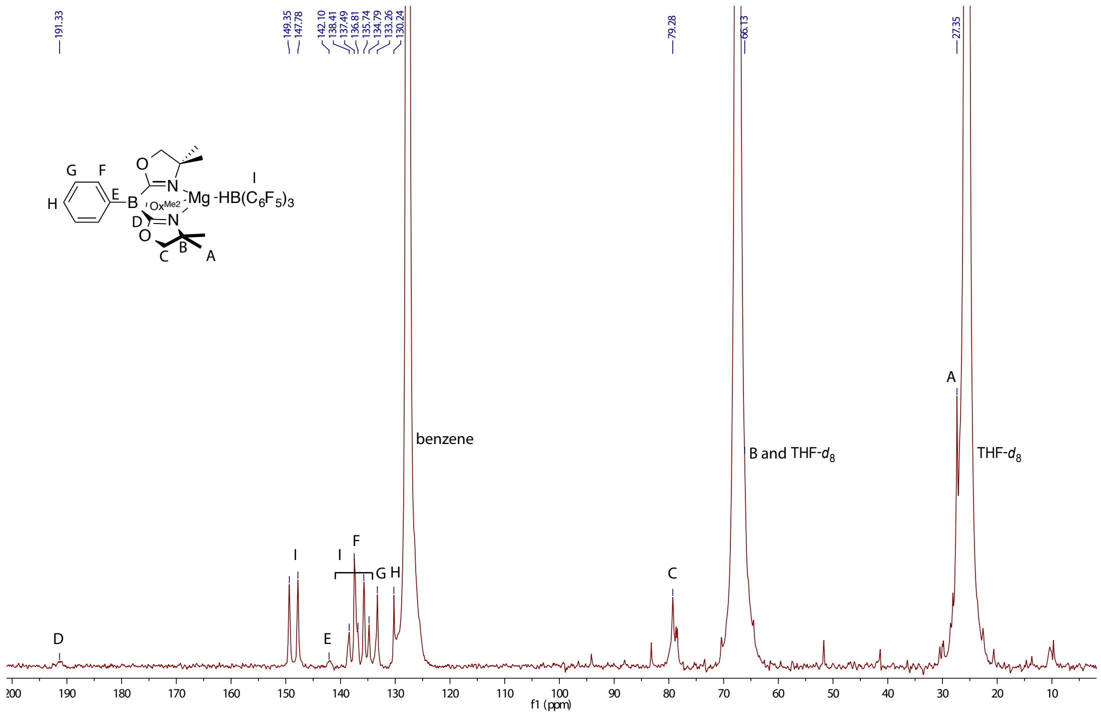


**Figure S2.** $^{13}C\{^{1}H\}$ NMR spectrum (150 MHz, THF-$d_8$) of To$^{M}$MgHB($C_6F_5$)$_3$.

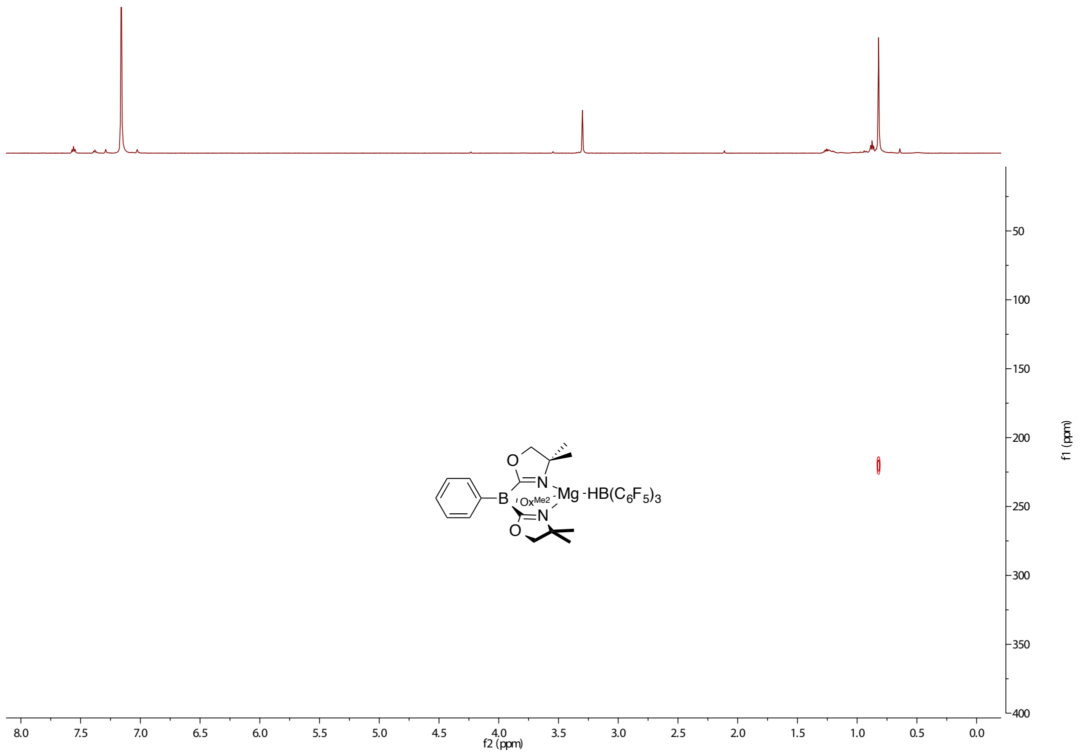


**Figure S3.** $^{1}H$-$^{15}N$ HMBC experiment (60 MHz, benzene-$d_6$) of To$^{M}$MgHB($C_6F_5$)$_3$.

**To$^M$MgMeB($C_6F_5$)$_3$ (2).** A pentane solution of $B(C_6F_5)_3$ (0.031 g, 0.059 mmol) was added to a concentrated solution of To$^M$MgMe (0.025 g, 0.059 mmol) in benzene. The reaction mixture was stirred for 15 min. and then allowed to stand for 30 min. as a white precipitate formed. The solvent was decanted, and the residue washed with pentane (3 × 5 mL) and then dried under vacuum to provide To$^M$MgMeB($C_6F_5$)$_3$ (0.046 g, 0.049 mmol, 83.2%). When generated in situ, To$^M$MgMeB($C_6F_5$)$_3$ is soluble in benzene-$d_6$ and $CD_2Cl_2$ and NMR spectra can be obtained, with the exception of $^{13}$C NMR spectral data due to the rate of conversion from To$^M$MgMeB($C_6F_5$)$_3$ to To$^M$Mg$C_6F_5$ being too fast. However, once solvent is removed and To$^M$MgMeB($C_6F_5$)$_3$ isolated, it becomes insoluble in benzene and methylene chloride and only partially redissolves in THF-$d_8$. Though benzene, methylene chloride, and THF solvents were attempted, $^{13}$C NMR spectral data could not be obtained due to the low solubility of To$^M$MgMeB($C_6F_5$)$_3$ and its spontaneous conversion to To$^M$Mg$C_6F_5$. $^1$H NMR (600 MHz, benzene-$d_6$): δ 0.79 (s, 18 H, CNC*Me*$_2$CH$_2$O), 1.27 (br s, 3 H, MgMeB($C_6F_5$)$_3$), 3.23 (s, 6 H, CNCMe$_2$C*H*$_2$O), 7.35 (br, 1 H, *para*-$C_6H_5$), 7.51 (br, 2 H, *meta*-$C_6H_5$), 8.09 (br, 2 H, *ortho*-$C_6H_5$). $^{11}$B NMR (192 MHz, benzene-$d_6$): δ –15.5 (Me*B*($C_6F_5$)$_3$), –18.5 (To$^M$). $^{19}$F NMR (544 MHz, benzene-$d_6$): δ –134.8 (*ortho*-$C_6F_5$), –159.1 (*para*-$C_6F_5$), –163.4 (*meta*-$C_6F_5$). $^{15}$N NMR (60 MHz, benzene-$d_6$): δ –162.2. IR (KBr, cm$^{-1}$): ν 3037 (w), 2973 (s), 2935 (m), 1636 (m), 1541 (s), 1508 (s), 1458 (s), 1437 (s), 1374 (m), 1267 (m), 1202 (m), 1088 (s), 965 (s), 842 (w), 803 (w), 738 (w), 683 (w). Anal. Calcd. for $C_{40}H_{32}B_2F_{15}MgN_3O_3$: C, 51.46; H, 3.45; N, 4.50. Found C, 51.09; H, 3.20; N, 4.01. Mp: 205-207 °C (dec.).

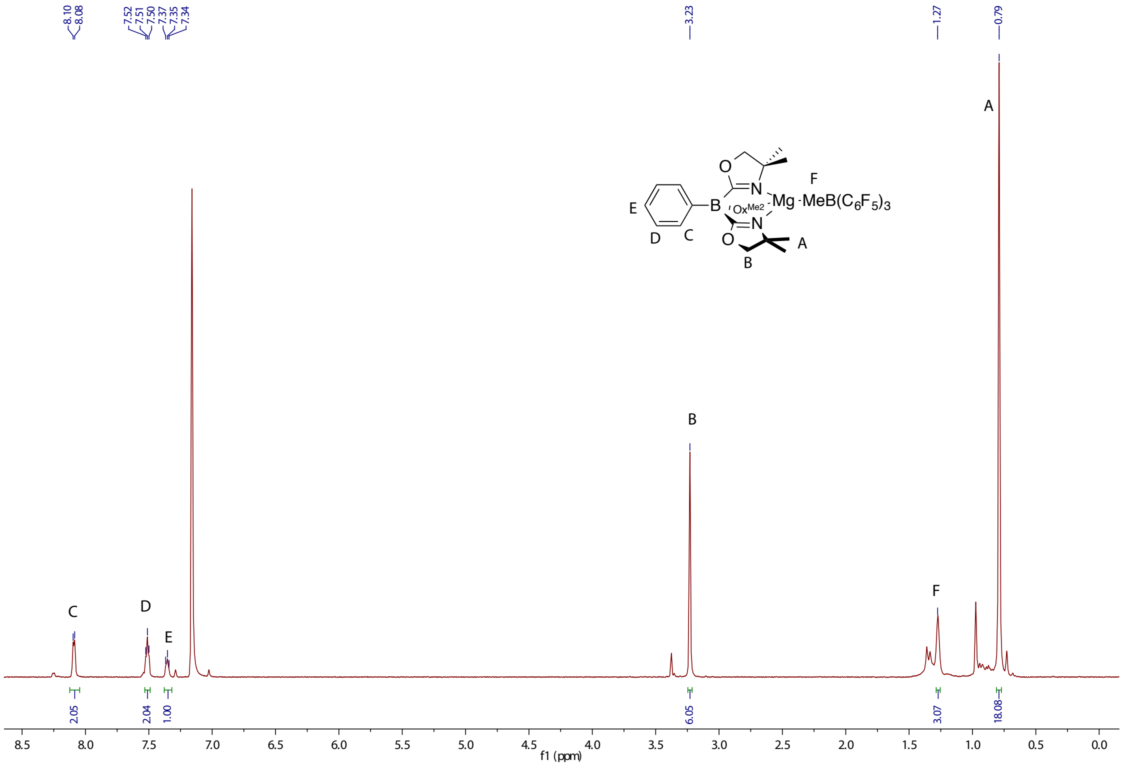


**Figure S4.** $^{1}H$ NMR spectrum (600 MHz, benzene-$d_6$) of $To^{M}MgMeB(C_6F_5)_3$.

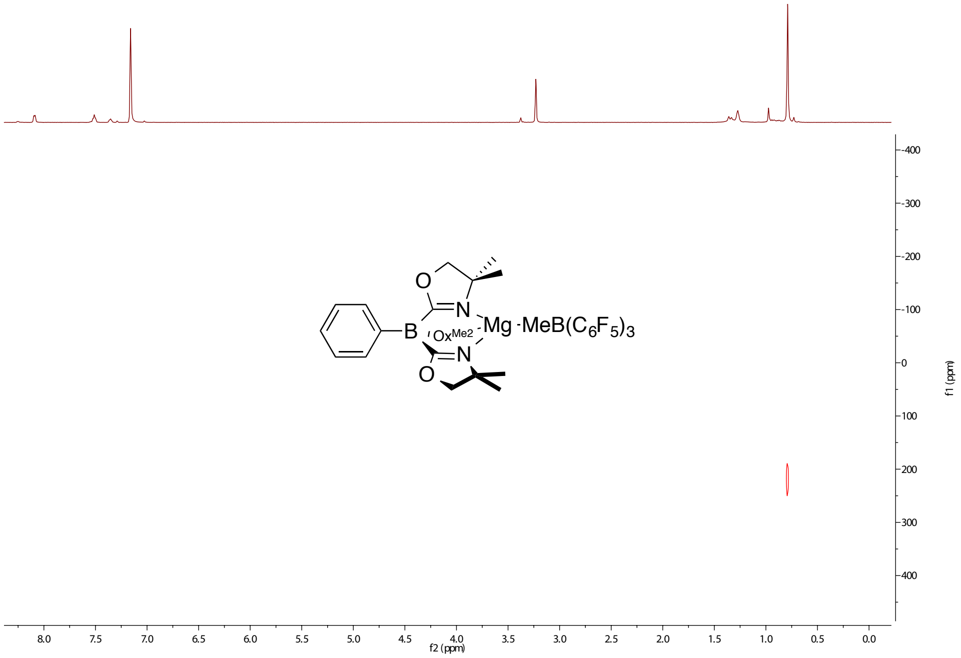


**Figure S5.** $^{1}H$-$^{15}N$ HMBC experiment (60 MHz, benzene-$d_6$) on $To^{M}MgMeB(C_6F_5)_3$.

**To$^{M}$MgC$_6$F$_5$.** B($C_6F_5$)$_3$ (0.024 g, 0.047 mmol) and 1 equiv. of To$^{M}$MgMe (0.020 g, 0.047 mmol) were dissolved in methylene chloride, and the reaction mixture was stirred for 24 h, and then filtered. Solvent was removed under reduced pressure. The white solid was washed with pentane (3 × 5 mL) to afford To$^{M}$MgC$_6$F$_5$ (0.012 g, 0.021 mmol, 44.4%). $^{1}$H NMR (600 MHz, benzene-$d_6$): δ 0.97 (s, 18 H, CNC*Me*$_2$CH$_2$O), 3.38 (s, 6 H, CNCMe$_2$C*H*$_2$O), 7.38 (m, $^{3}J_{HH}$ = 7.2 Hz, 1 H, *para*-$C_6H_5$), 7.55 (m, $^{3}J_{HH}$ = 7.6 Hz, 2 H, *meta*-$C_6H_5$), 8.25 (d, $^{3}J_{HH}$ = 7.2 Hz, 2 H, *ortho*-$C_6H_5$). $^{13}$C{$^{1}$H} NMR (150 MHz, benzene-$d_6$): δ 28.08 (CNC*Me*$_2$CH$_2$O), 65.91 (CN*C*Me$_2$CH$_2$O), 80.81 (CNCMe$_2$*C*H$_2$O), *para*-$C_6H_5$ and *meta*-$C_6H_5$ overlapping with benzene-$d_6$, 136.41 (*ortho*-$C_6H_5$), $C_6F_5$ too low intensity to be observed, 148.29 (br, *ipso*-$C_6H_5$), 193.08 (br, *C*NCMe$_2$CH$_2$O). $^{11}$B NMR (192 MHz, benzene-$d_6$): δ –18.3. $^{19}$F NMR (376 MHz, benzene-$d_6$): δ –110.5 (2 F, *ortho*-$C_6F_5$), –155.8 (1 F, *para*-$C_6F_5$), –160.7 (2 F, *meta*-$C_6F_5$). $^{15}$N NMR (60 MHz, benzene-$d_6$): δ –157.7. IR (KBr, cm$^{-1}$): ν 2965 (s), 2926 (s), 2884 (m), 2853 (m), 1594 (s, CN), 1510 (m), 1463 (s), 1366 (m), 1350 (w), 1268 (s), 1197 (m), 1152 (m), 1117 (w), 1086 (m), 967 (s), 892 (w), 839 (w), 810 (m), 746 (w), 703 (m). Anal. Calcd. for $C_{27}H_{29}BF_5MgN_3O_3$: C, 56.53; H, 5.10; N, 7.33. Found C, 57.01; H, 5.58; N, 6.89. Mp: 195-197 °C.

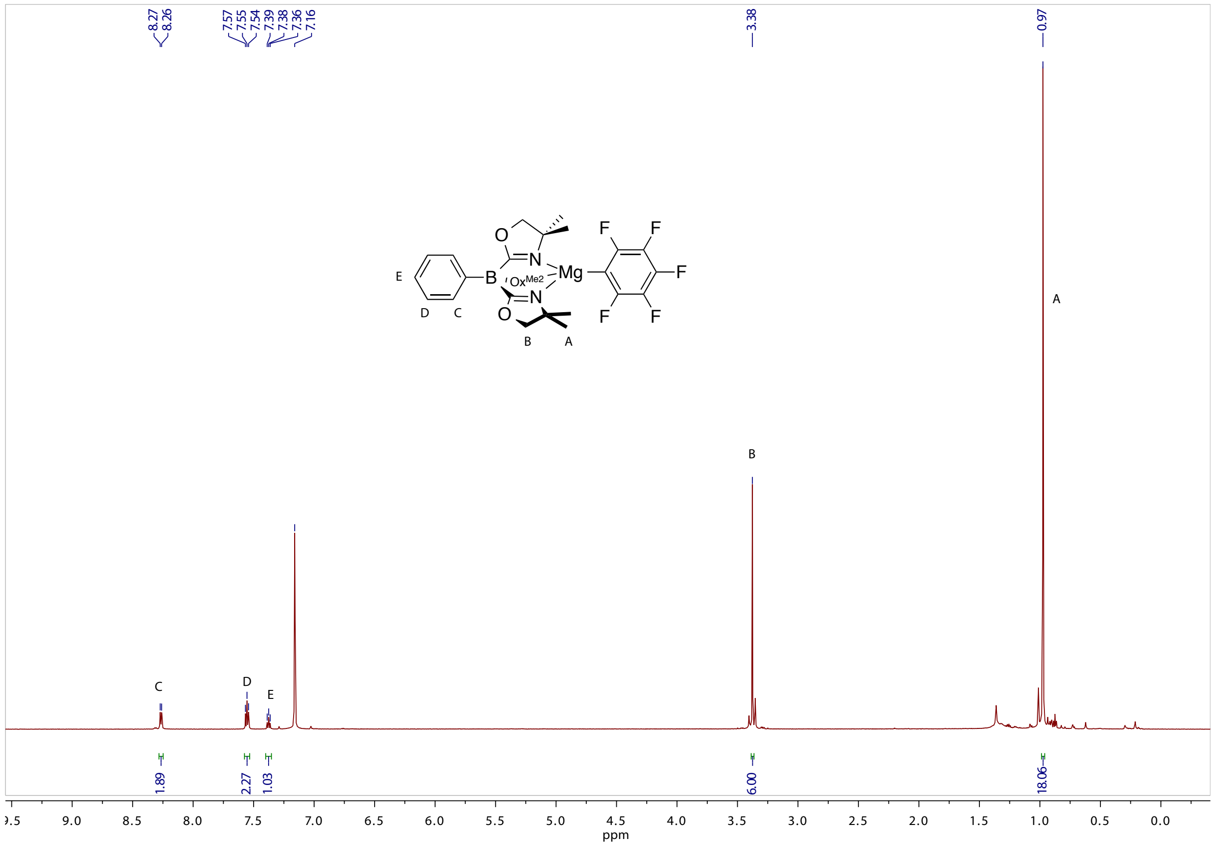


**Figure S6.** $^{1}H$ NMR spectrum (600 MHz, benzene-$d_6$) of $To^{M}MgC_6F_5$.

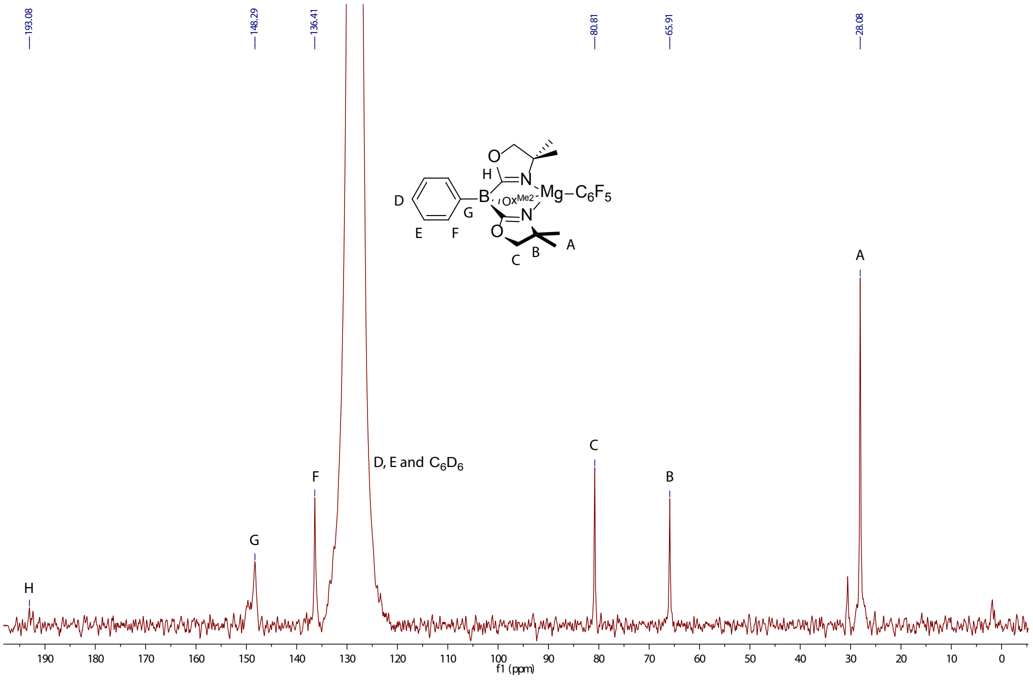


**Figure S7.** $^{13}C$ NMR spectrum (150 MHz, benzene-$d_6$) of $To^{M}MgC_6F_5$.

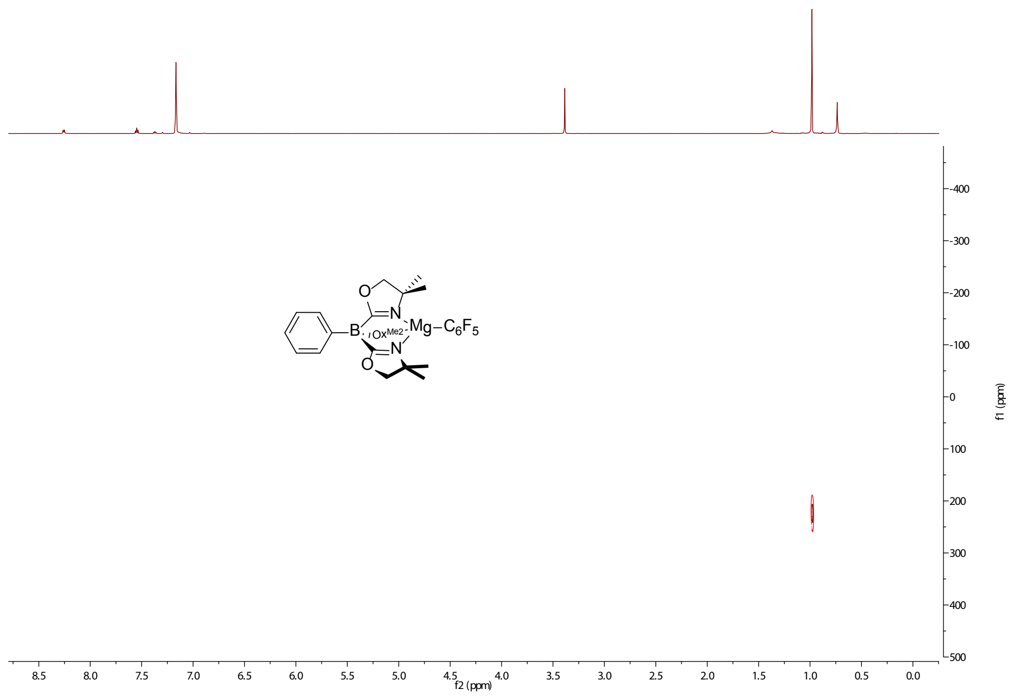

**Figure S8.** $^{1}H$-$^{15}N$ NMR HMBC experiment (60 MHz, benzene-$d_6$) on $To^{M}MgC_6F_5$.

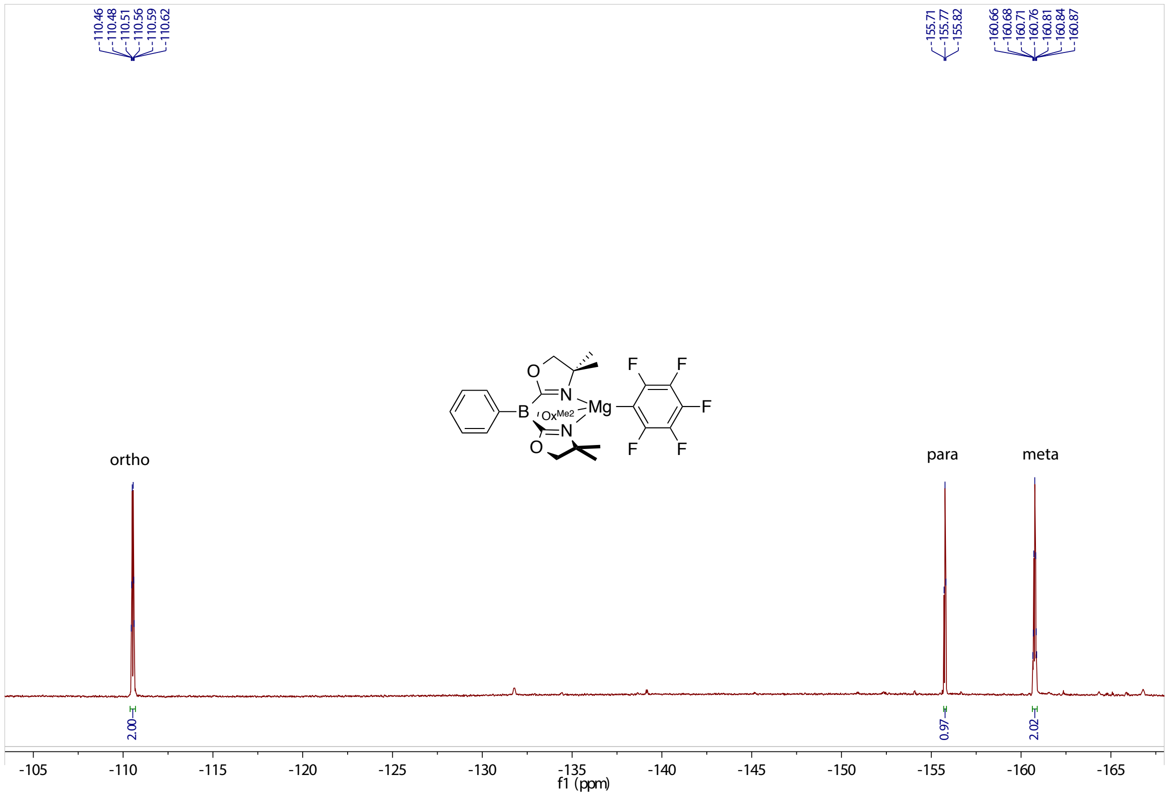


**Figure S9.** $^{19}F$ NMR spectrum of $To^{M}MgC_6F_5$.

**[To$^M$Mg][B($C_6F_5$)$_4$].** To$^M$MgMe (0.026 g, 0.062 mmol) and [$Ph_3C$][B($C_6F_5$)$_4$] (0.057 g, 0.062 mmol) were dissolved in benzene (10 mL) and stirred for 30 min. to obtain a white precipitate, which was then isolated by decantation of the top liquid. Washing with benzene (3×5 mL) followed by drying under vacuum provided [To$^M$Mg][B($C_6F_5$)$_4$] (0.064 g, 0.059 mmol, 94.9%). Solution-phase characterization is given in THF-$d_8$ of the THF-$d_8$ adduct. $^1$H NMR (600 MHz, THF-$d_8$): δ 1.28 (s, 18 H, CNC$Me_2$$CH_2$O), 3.90 (s, 6 H, CNCMe$_2$C$H_2$O), 7.01 (br, 1 H, *para*-$C_6H_5$), 7.07 (br, 2 H, *meta*-$C_6H_5$), 7.64 (br, 2 H, *ortho*-$C_6H_5$). $^{13}$C{$^1$H} NMR (150 MHz, THF-$d_8$): δ 28.61 (CNC$Me_2$$CH_2$O), 66.37 (CN*C*Me$_2$CH$_2$O), 80.49 (CNCMe$_2$*C*H$_2$O), 125.33 (*para*-$C_6H_5$), 126.56 (*meta*-$C_6H_5$), 134.5 (br, *ipso*-$C_6H_5$), 136.00 (*ortho*-$C_6H_5$), 136.38 ($C_6F_5$), 138.02 ($C_6F_5$), 138.41 ($C_6F_5$), 140.02 ($C_6F_5$), 148.43 ($C_6F_5$), 150.07 ($C_6F_5$), 191 (br, *C*NCMe$_2$CH$_2$O). $^{11}$B NMR (192 MHz, THF-$d_8$): δ –18.4, –20.2 (B($C_6F_5$)$_4$). $^{19}$F NMR (544 MHz, THF-$d_8$): δ –134.6 (*ortho*-$C_6F_5$), –166.7 (*para*-$C_6F_5$), –170.3 (*meta*-$C_6F_5$). $^{15}$N NMR (THF-$d_8$, 60 MHz): δ –156. IR (KBr, cm$^{-1}$): *v* 2981 (s), 1645 (m), 1559 (w), 1515 (s), 1464 (s), 1374 (w), 1277 (m), 1089 (s), 980 (s), 862 (s), 775 (m), 756 (m), 684 (m), 661 (m). Anal. Calcd. for $C_{45}H_{29}B_2F_{20}MgN_3O_3$: C, 49.79; H, 2.69; N, 3.87. Found C, 50.20; H, 2.49; N, 4.01. Mp: 86-88 °C.

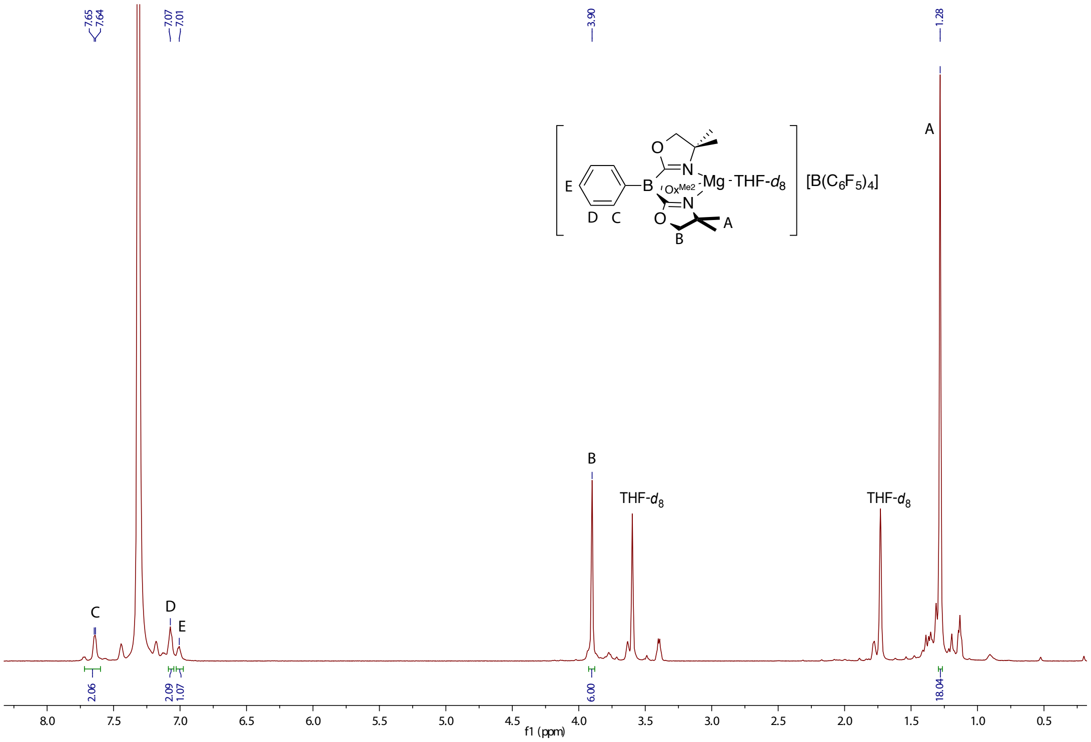


**Figure S10.** $^1$H NMR spectrum (600 MHz, THF-$d_8$) of [To$^M$Mg][B($C_6F_5$)$_4$].

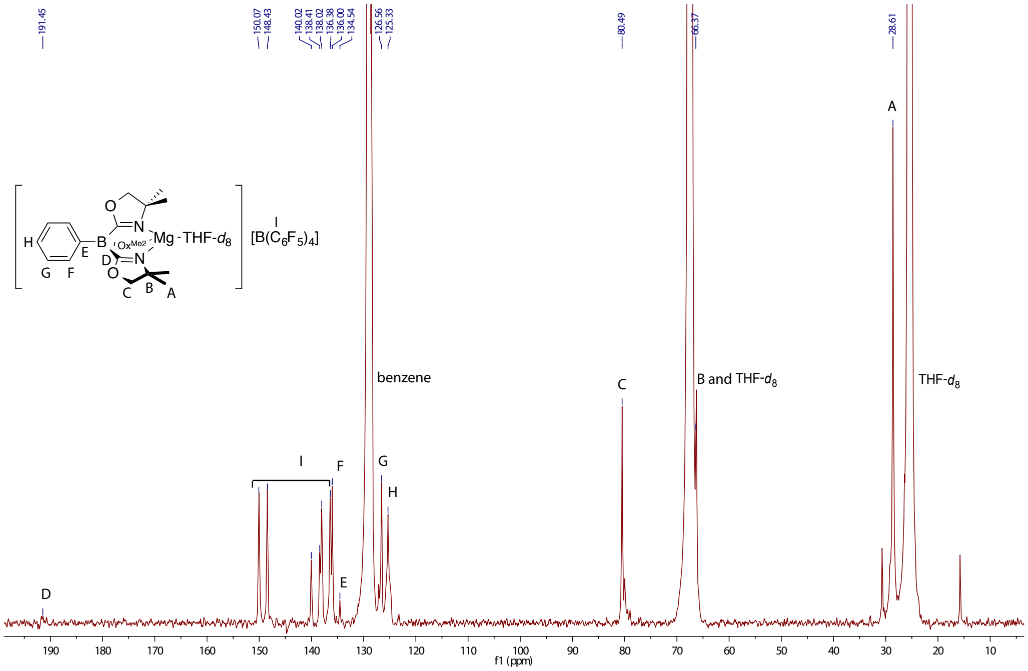


**Figure S11.** $^{13}$C NMR spectrum (150 MHz, THF-$d_8$) of [To$^M$Mg][B($C_6F_5$)$_4$].

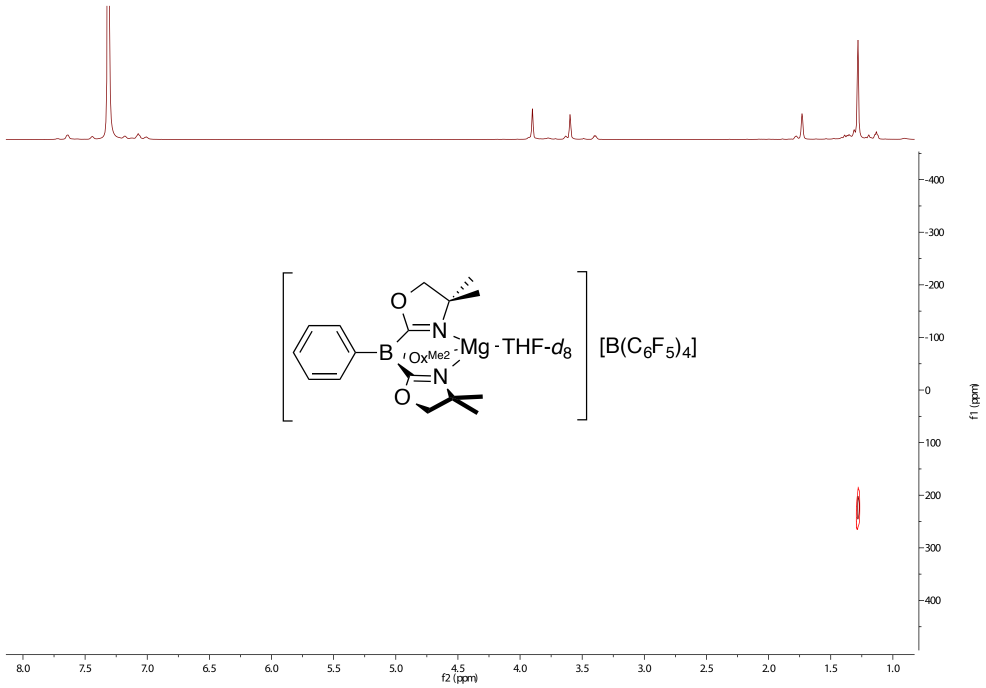


**Figure S12.** $^{1}H$-$^{15}N$ NMR experiment (60 MHz, THF-$d_8$) on [To$^{M}$Mg][B($C_6F_5$)$_4$].

**BnMe$_2$SiO(MeO)C=CMe$_2$.** Crystallized To$^{M}$MgHB($C_6F_5$)$_3$ (0.011 g, 0.012 mmol), methyl methacrylate (0.117 g, 1.17 mmol), and BnMe$_2$SiH (0.176 g, 1.17 mmol) were stirred in benzene for 30 min. at room temperature. Evaporation of the volatile materials under reduced pressure afforded a colorless gel. The product was extracted with pentane, and the extracts were evaporated under reduced pressure to afford a colorless liquid (0.290 g, 1.16 mmol, 99.0%). $^{1}H$ NMR (600 MHz, benzene-$d_6$): δ 0.15 (s, 6 H, SiMe$_2$), 1.61 (s, 3 H, C=C*Me*$_2$), 1.71 (s, 3 H, C=C*Me*$_2$), 2.23 (s, 2 H, PhC*H*$_2$Si), 3.29 (s, 3 H, OMe), 7.02 (m, 3 H, $C_6H_5$), 7.13 (m, 2 H, $C_6H_5$). $^{13}C\{^{1}H\}$ NMR (150 MHz, benzene-$d_6$): δ –1.44 (SiMe$_2$), 16.84 (C=C*Me*$_2$), 17.49 (C=C*Me*$_2$), 27.30 (Ph*C*H$_2$Si), 57.01 (OMe), 91.41 (C=*C*Me$_2$), 125.14 ($C_6H_5$), 128.98 ($C_6H_5$), 129.20 ($C_6H_5$), 139.10 (*ipso*-$C_6H_5$), 150.64 (*C*=CMe$_2$). $^{29}Si$ (119 MHz, benzene-$d_6$) δ 15.7. IR (KBr, cm$^{-1}$): *ν* 3062 (w), 3027 (m), 2964 (s), 2919 (s), 2859 (s), 1706 (s), 1601 (m), 1494 (m), 1453 (m), 1410

(m), 1256 (s), 1203 (s), 1176 (s), 1058 (w), 1027 (s), 944 (s), 905 (w), 862 (s), 763 (s), 699 (s), 664 (w), 611 (w). Anal. Calcd. for $C_{14}H_{22}O_2Si$: C, 67.15; H, 8.86. Found C, 67.63; H, 8.73.

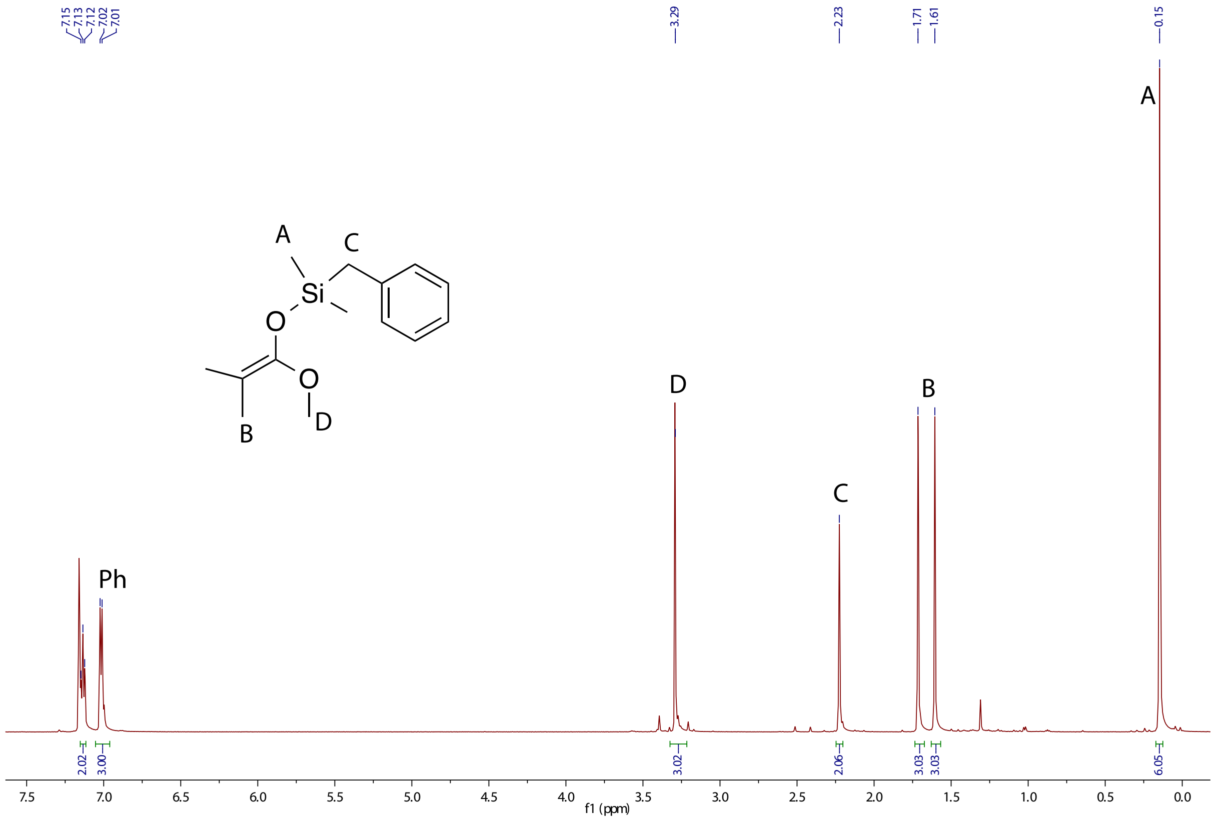


**Figure S13.** $^1$H NMR spectrum (600 MHz, benzene-$d_6$) of $BnMe_2SiO(MeO)C{=}CMe_2$.

**$(H_2C{=}CH)Me_2SiO(MeO)C{=}CMe_2$.** Crystallized $To^MMgHB(C_6F_5)_3$ (0.011 g, 0.012 mmol), methyl methacrylate (0.117 g, 1.17 mmol), and $(CH_2{=}CH)Me_2SiH$ (0.101 g, 1.17 mmol) were stirred in benzene for 30 min. at room temperature. The volatile materials were removed under reduced pressure, leaving behind a colorless gel. The product was extracted with pentane, and the extracts were evaporated under reduced pressure to afford a colorless liquid (0.201 g, 1.08 mmol, 92.1%). $^1$H NMR (400 MHz, benzene-$d_6$): δ 0.26 (s, 6 H, $Me_2Si$), 1.67 (s, 3 H, C=C*Me*$_2$), 1.73 (s, 3 H, C=C*Me*$_2$), 3.34 (s, 3 H, OMe), 5.77 (dd, 1 H, $^3J_{HH}$ = 21 Hz, $^2J_{HH}$ = 3.6 Hz,

$H_2$C=CHSi), 5.91 (dd, 1 H, $^3J_{HH}$ = 15 Hz, $^2J_{HH}$ = 3.6 Hz, $H_2$C=CHSi), 6.22 (dd, 1 H, $^3J_{HH}$ = 15 Hz, $^2J_{HH}$ = 16 Hz, $H_2$C=C$H$Si). $^{13}$C{$^1$H} NMR (150 MHz, benzene-$d_6$): δ –1.20 ($Me_2$Si), 16.84 (C=C$Me_2$), 17.57 (C=C$Me_2$), 56.90 (OMe), 91.01 (C=$C$Me$_2$), 133.61 ($H_2C$=CHSi), 137.10 ($H_2$C=$C$HSi), 150.59 ($C$=CMe$_2$). $^{29}$Si (119 MHz, benzene-$d_6$) δ 17.5. IR (KBr, cm$^{-1}$): $v$ 3055 (m), 2972 (s), 1716 (s), 1605 (w), 1567 (w), 1414 (m), 1266 (s), 1166 (s), 1029 (m), 955 (m), 900 (w), 836 (s), 790 (s), 744 (w), 717 (w), 690 (m), 662 (m). Anal. Calcd. for $C_9H_{18}O_2Si$: C, 58.02; H, 9.74. Found C, 58.50; H, 9.62.

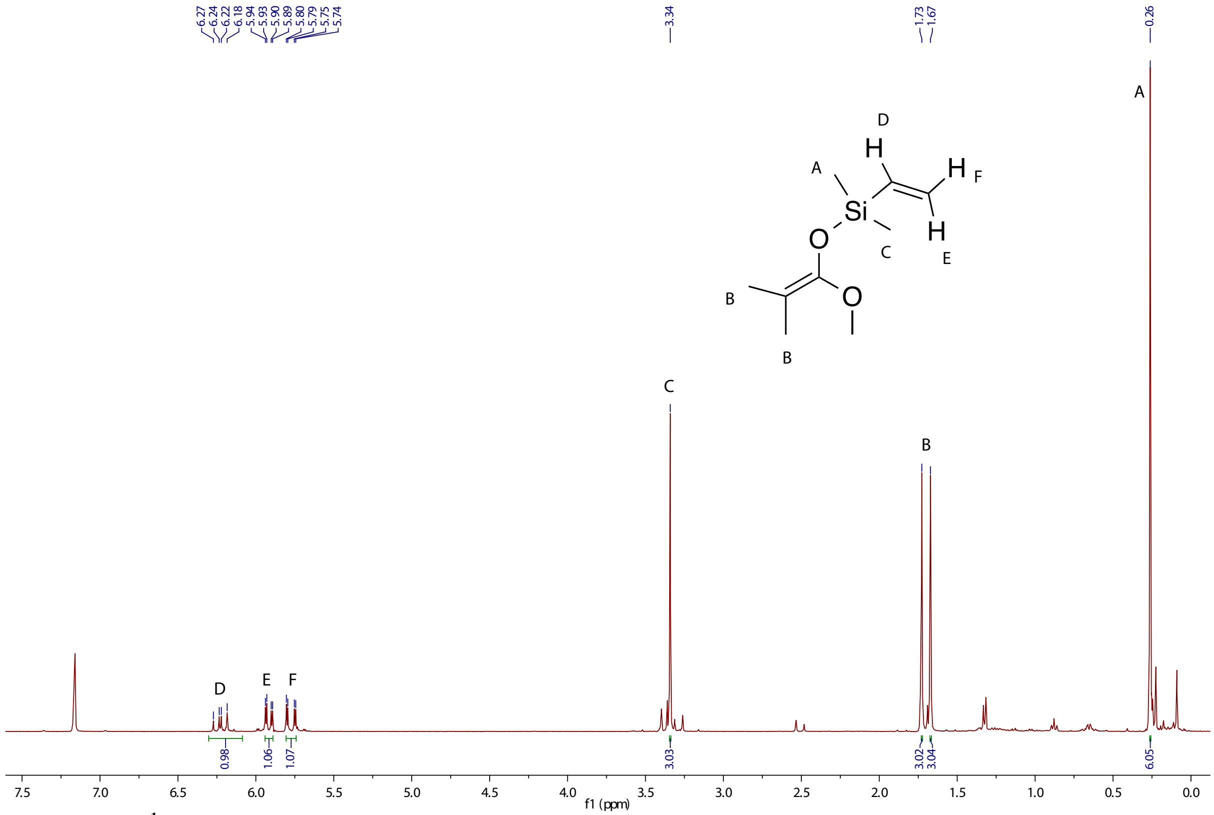


**Figure S14.** $^1$H NMR spectrum (400 MHz, benzene-$d_6$) of ($H_2$C=CH)Me$_2$SiO(MeO)C=CMe$_2$.

**Ph$_2$HSiO(MeO)C=CMe$_2$.** Crystallized To$^M$MgHB($C_6F_5$)$_3$ (0.011 g, 0.012 mmol), methyl methacrylate (0.117 g, 1.17 mmol), and Ph$_2$SiH$_2$ (0.216 g, 1.17 mmol) were stirred in benzene for 30 min. at room temperature. Benzene was removed under reduced pressure, leaving behind a

colorless gel. The product was extracted with pentane, and the extracts were evaporated under reduced pressure to afford a colorless liquid (0.331 g, 1.13 mmol, 96.3%). $^1$H NMR (400 MHz, benzene-$d_6$): δ 1.64 (s, 3 H, C=C*Me*$_2$), 1.69 (s, 3 H, C=C*Me*$_2$), 3.29 (s, 3 H, OMe), 5.84 (s, 1 H, SiH), 7.17 (m, 6 H, $C_6H_5$), 7.74 (m, 4 H, $C_6H_5$). $^{13}$C{$^1$H} NMR (150 MHz, benzene-$d_6$): δ 16.79 (C=C*Me*$_2$), 17.42 (C=C*Me*$_2$), 57.92 (OMe), 91.74 (C=*C*Me$_2$), 130.47 ($C_6H_5$), 131.12 ($C_6H_5$), 134.16 (*ipso*-$C_6H_5$), 135.49 ($C_6H_5$), 136.39 ($C_6H_5$), 150.93 (*C*=CMe$_2$). $^{29}$Si (119 MHz, benzene-$d_6$) δ –14.5 (d, $^1J_{SiH}$ = 201 Hz). IR (KBr, cm$^{-1}$): $\nu$ 3094 (m), 2931 (s), 2158 (s), 1716 (s), 1661 (w), 1598 (m), 1566 (w), 1548 (w), 1528 (w), 1437 (s), 1263 (m), 1169 (br s), 1029 (m), 949 (m), 858 (s), 738 (s), 701 (s), 671 (w). Anal. Calcd. for $C_{17}H_{20}O_2Si$: C, 71.79; H, 7.09. Found C, 71.61; H, 7.32.

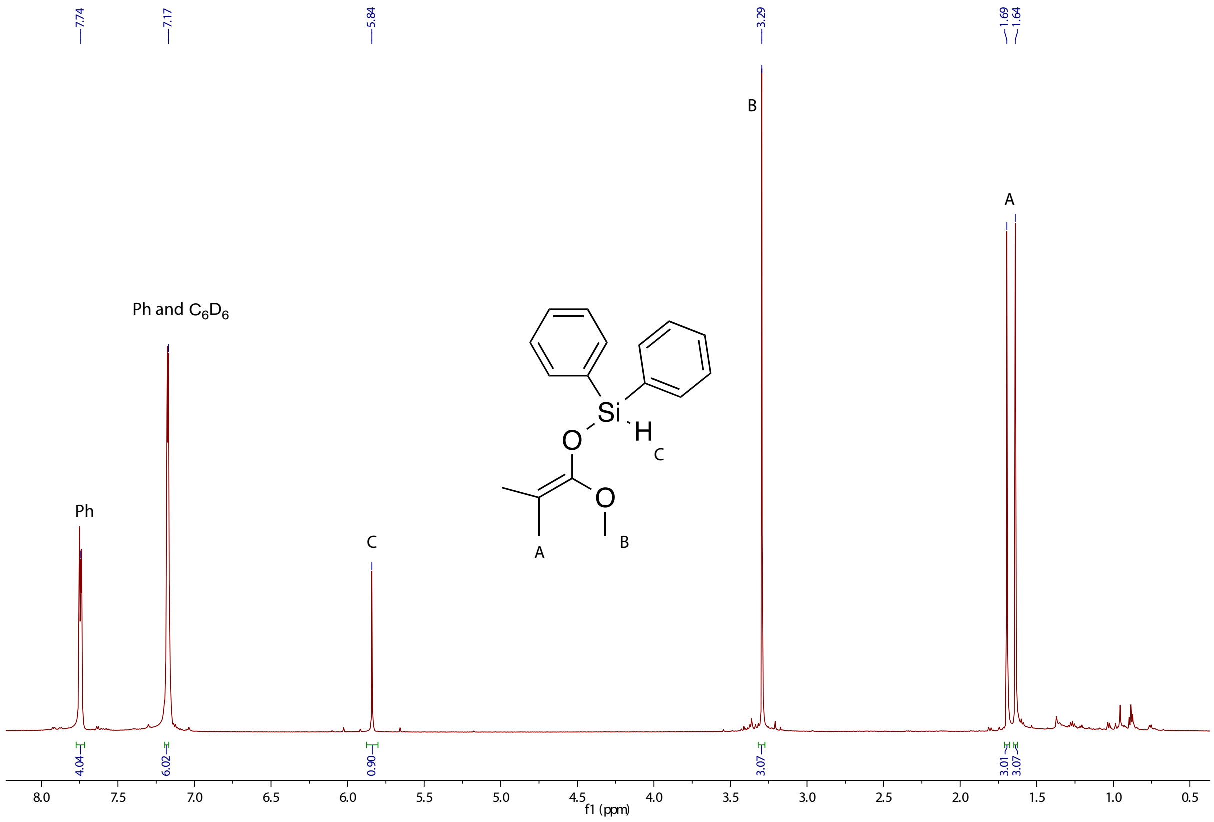


**Figure S15.** $^1$H NMR spectrum (400 MHz, benzene-$d_6$) of $Ph_2HSiO(MeO)C{=}CMe_2$.

**PhMeHSiO(MeO)C=CMe$_2$.** Crystallized To$^M$MgHB($C_6F_5$)$_3$ (0.0428 g, 0.047 mmol), methyl methacrylate (0.467 g, 4.66 mmol), and PhMeSiH$_2$ (0.570 g, 4.66 mmol) were stirred in benzene for 7 h at 60 °C. The volatile materials were evaporated under reduced pressure to give a colorless gel. The product was extracted with pentane, and the extracts were evaporated under reduced pressure. Repeated Kugelrohr distillation (3×) under dynamic vacuum at 170 °C provided a colorless liquid (1.01 g, 4.54 mmol, 97.1%). $^1$H NMR (600 MHz, benzene-$d_6$): δ 0.44 (d, 3 H, $^3J_{HH}$ = 2.8 Hz, SiMe), 1.66 (s, 3 H, C=C*Me*$_2$), 1.67 (s, 3 H, C=C*Me*$_2$), 3.30 (s, 3 H, OMe), 5.36 (q, 1 H, $^3J_{HH}$ = 2.8 Hz, SiH), 7.18 (m, 3 H, $C_6H_5$), 7.64 (m, 2 H, $C_6H_5$). $^{13}$C{$^1$H} NMR (150 MHz, benzene-$d_6$): δ –2.31 (SiMe), 16.78 (C=C*Me*$_2$), 17.36 (C=C*Me*$_2$), 57.41 (OMe), 91.31 (C=*C*Me$_2$), 128.63 ($C_6H_5$), 130.92 ($C_6H_5$), 134.61 ($C_6H_5$), 135.73 (*ipso*-$C_6H_5$), 150.75 (*C*=CMe$_2$). $^{29}$Si (119 MHz, benzene-$d_6$) δ –3.8 (d, $^1J_{SiH}$ = 216 Hz). IR (KBr, cm$^{-1}$): $\nu$ 3062 (m), 2897 (s), 2809 (m), 2076 (s, $\nu_{SiH}$), 1740 (s), 1589 (w), 1451 (m), 1369 (w), 1257 (s), 1178 (s), 863 (s), 802 (w), 775 (m), 741 (m), 705 (m). Anal. Calcd. for $C_{12}H_{18}O_2Si$: C, 64.82; H, 8.16. Found C, 64.85; H, 7.79.

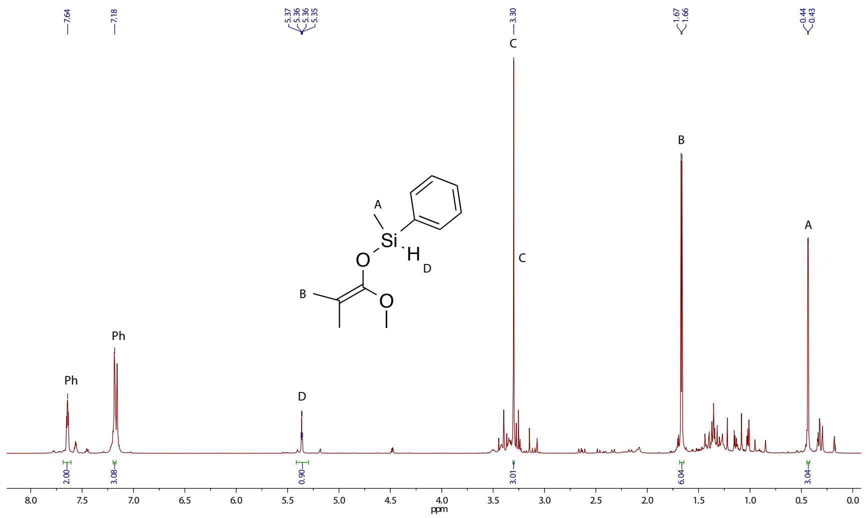


**Figure S16.** $^{1}H$ NMR spectrum (600 MHz, benzene-$d_6$) of $PhMeHSiO(MeO)C{=}CMe_2$, obtained from hydrosilylation catalyzed by **1**.

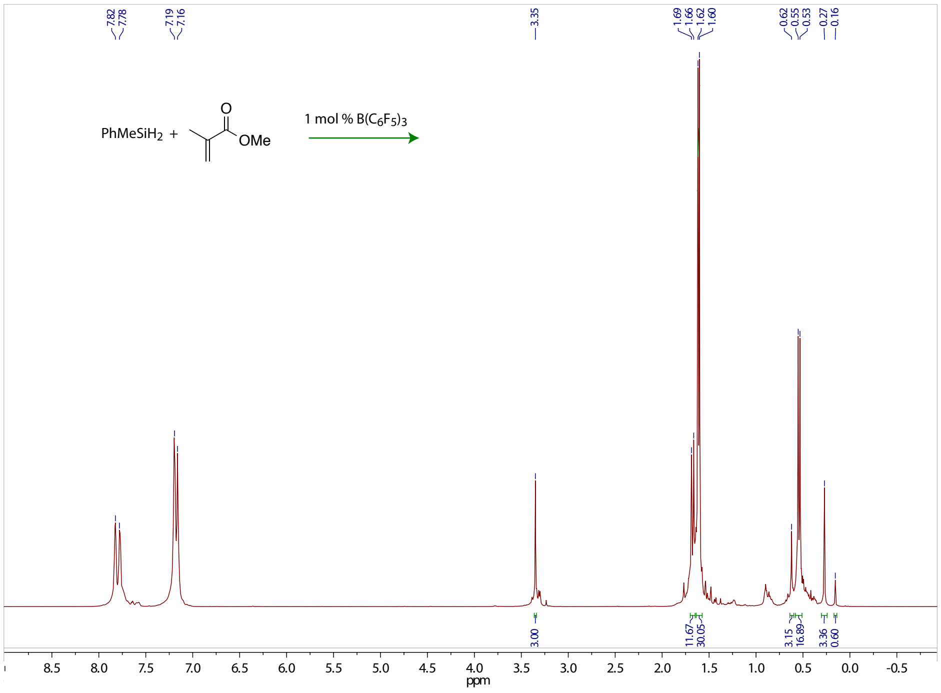


**Figure S17.** $^{1}H$ NMR spectrum of the reaction of $PhMeSiH_2$ and methyl methacrylate, catalyzed by 1 mol % $B(C_6F_5)_3$, which gives a mixture of bis-addition products and ester reductive cleavage products, distinct from the reactions catalyzed by $To^{M}MgHB(C_6F_5)_3$ (Figure S16). See Figure S23 for authentic $PhMeSi\{OC(OMe){=}CMe_2\}_2$ from the selective $To^{M}MgHB(C_6F_5)_3$ catalyzed addition.

**$PhMeHSiO(Me_2CHCH_2O)C{=}CMe_2$.** Crystallized $To^MMgHB(C_6F_5)_3$ (0.027 g, 0.029 mmol), isobutyl methacrylate (0.167 g, 1.17 mmol), and $PhMeSiH_2$ (0.149 g, 1.17 mmol) were stirred in benzene for 8 h at room temperature. The solvent was evaporated under reduced pressure, leaving behind a colorless gel. The product was extracted with pentane, and the extracts were evaporated to dryness under reduced pressure to provide a colorless liquid (0.126 g, 0.476 mmol, 40.5%). $^1$H NMR (600 MHz, benzene-$d_6$): δ 0.46 (d, 3 H, $^3J_{HH}$ = 3.0 Hz, SiMe), 0.85 (d, 3 H, $^3J_{HH}$ = 6.6 Hz, $Me_2CHCH_2O$), 0.86 (d, 3 H, $^3J_{HH}$ = 6.6 Hz, $Me_2CHCH_2O$), 1.69 (s, 3 H, C=C*Me*$_2$), 1.72 (s, 3 H, C=C*Me*$_2$), 1.81 (m, 1 H, $Me_2CHCH_2O$), 3.49 (d, 2 H, $^3J_{HH}$ = 6.6 Hz, $Me_2CHCH_2O$), 5.39 (q, 1 H, $^3J_{HH}$ = 3.0 Hz, SiH), 7.18 (m, 3 H, $C_6H_5$), 7.66 (m, 2 H, $C_6H_5$). $^{13}C\{^1H\}$ NMR (150 MHz, benzene-$d_6$): δ –2.26 (SiMe), 16.96 (C=C*Me*$_2$), 17.50 (C=C*Me*$_2$), 19.75 ($OCH_2CHMe_2$), 29.20 ($Me_2CHCH_2O$), 76.78 ($Me_2CHCH_2O$), 91.51 (C=*C*Me$_2$), 128.62 ($C_6H_5$), 130.92 ($C_6H_5$), 134.62 ($C_6H_5$), 135.52 (*ipso*-$C_6H_5$), 149.96 (*C*=CMe$_2$). $^{29}$Si (119 MHz, benzene-$d_6$) δ –3.9 (d, $^1J_{SiH}$ = 222 Hz). IR (KBr, cm$^{-1}$): *ν* 2962 (s), 2926 (s), 2876 (s), 2141 (s), 1708 (s), 1470 (m), 1429 (m), 1393 (w), 1257 (s), 1166 (s), 1122 (s), 1024 (m), 969 (w), 868 (s), 732 (w), 700 (m), 611 (m). Anal. Calcd. for $C_{15}H_{24}O_2Si$: C, 68.13; H, 9.15. Found C, 68.06; H, 8.69.

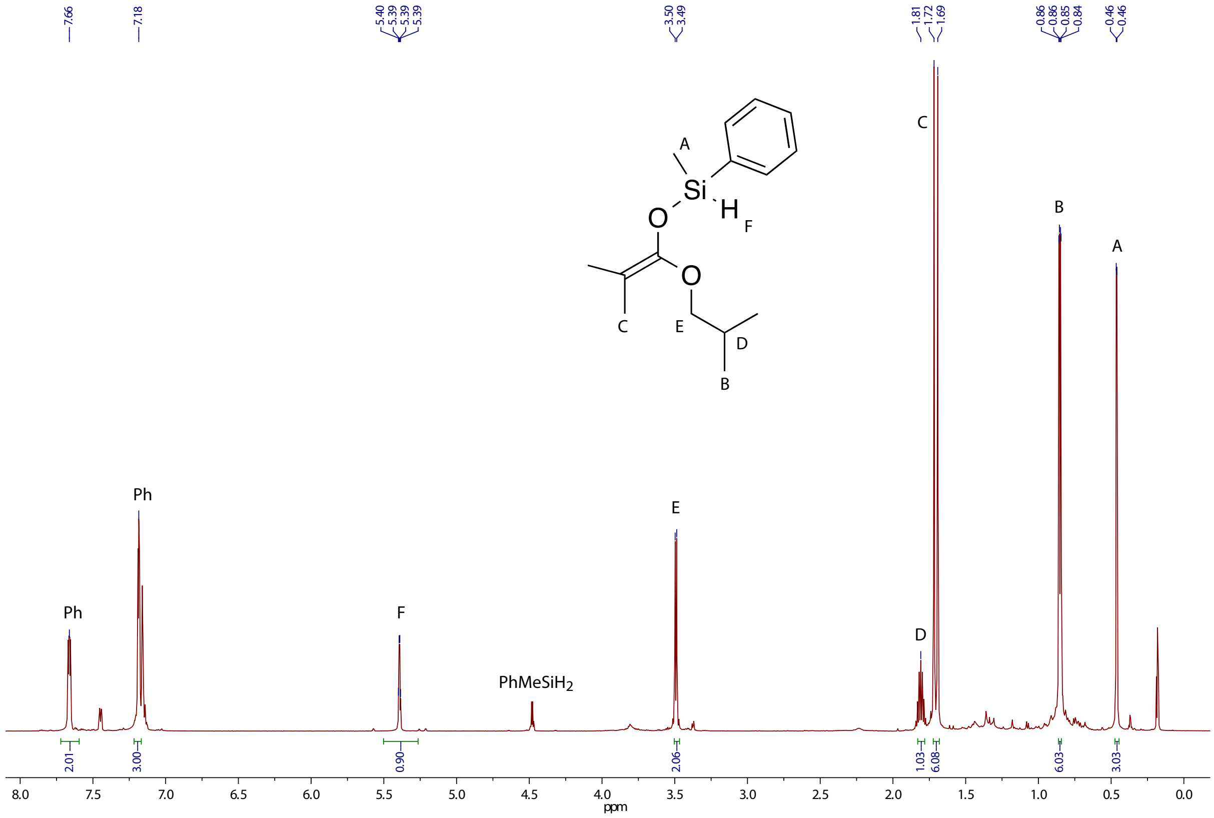


**Figure S18.** $^1$H NMR spectrum (600 MHz, benzene-$d_6$) of (PhMeHSiO)($Me_2CHCH_2O$)C=$CMe_2$.

**PhMeHSiO($PhCH_2O$)C=$CMe_2$.** Crystallized $To^MMgHB(C_6F_5)_3$ (0.027 g, 0.029 mmol), benzyl methacrylate (0.207 g, 1.17 mmol), and $PhMeSiH_2$ (0.149 g, 1.17 mmol) were stirred in benzene for 4 h at room temperature. The volatile materials were evaporated under reduced pressure to give a colorless gel. The product was extracted with pentane, and the extracts were evaporated under reduced pressure to provide a colorless liquid (0.348 g, 1.16 mmol, 99.2%). $^1$H NMR (600 MHz, benzene-$d_6$): δ 0.45 (d, 3 H, $^3J_{HH}$ = 3.0 Hz, SiMe), 1.62 (s, 3 H, C=C$Me_2$), 1.66 (s, 3 H, C=C$Me_2$), 4.70 (s, 2 H, PhC$H_2$O), 5.40 (q, 1 H, $^3J_{HH}$ = 3.0 Hz, SiH), 7.08 (t, 1 H, $^3J_{HH}$ = 7.2 Hz, $OCH_2C_6H_5$), 7.13 (t, 2 H, $^3J_{HH}$ = 7.2 Hz, $OCH_2C_6H_5$), 7.17 (m, 3 H, $SiC_6H_5$), 7.25 (d, 2 H, $^3J_{HH}$ = 7.2 Hz, $OCH_2C_6H_5$), 7.66 (m, 2 H, $SiC_6H_5$). $^{13}C\{^1H\}$ NMR (150 MHz, benzene-$d_6$): δ –2.37 (SiMe), 17.01 (C=C$Me_2$), 17.44 (C=C$Me_2$), 71.96 (O$CH_2$Ph), 92.83 (C=$C$Me$_2$), 128.35

(OCH$_2$*C*$_6$H$_5$), 128.66 (SiC$_6$H$_5$), 128.81 (OCH$_2$*C*$_6$H$_5$), 128.84 (OCH$_2$*C*$_6$H$_5$), 130.97 (SiC$_6$H$_5$), 134.61 (SiC$_6$H$_5$), 135.66 (*ipso*-SiC$_6$H$_5$), 149.22 (*C*=CMe$_2$). $^{29}$Si (119 MHz, benzene-*d*$_6$) δ –3.5 (d, $^1J_{SiH}$ = 216 Hz). IR (KBr, cm$^{-1}$): *v* 3070 (m), 3032 (m), 2982 (m), 2915 (s), 2859 (m), 2146 (s), 1707 (s), 1591 (w), 1498 (w), 1455 (m), 1429 (m), 1384 (w), 1256 (s), 1161 (s), 1122 (s), 1008 (m), 964 (m), 866 (s), 734 (s), 698 (s). Anal. Calcd. for $C_{18}H_{22}O_2Si$: C, 72.44; H, 7.43. Found C, 71.98; H, 7.49.

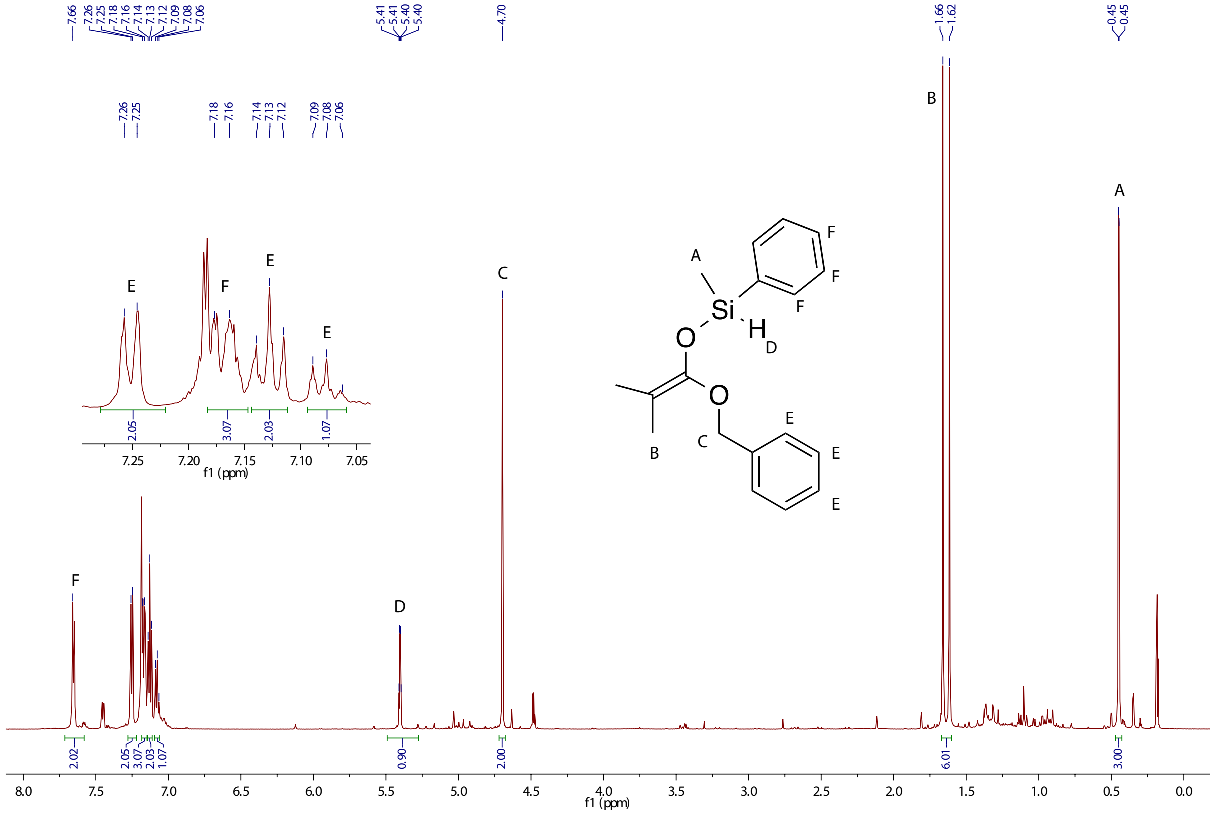


**Figure S19.** $^1$H NMR spectrum (600 MHz, benzene-*d*$_6$) of PhMeHSiO(PhCH$_2$O)C=CMe$_2$.

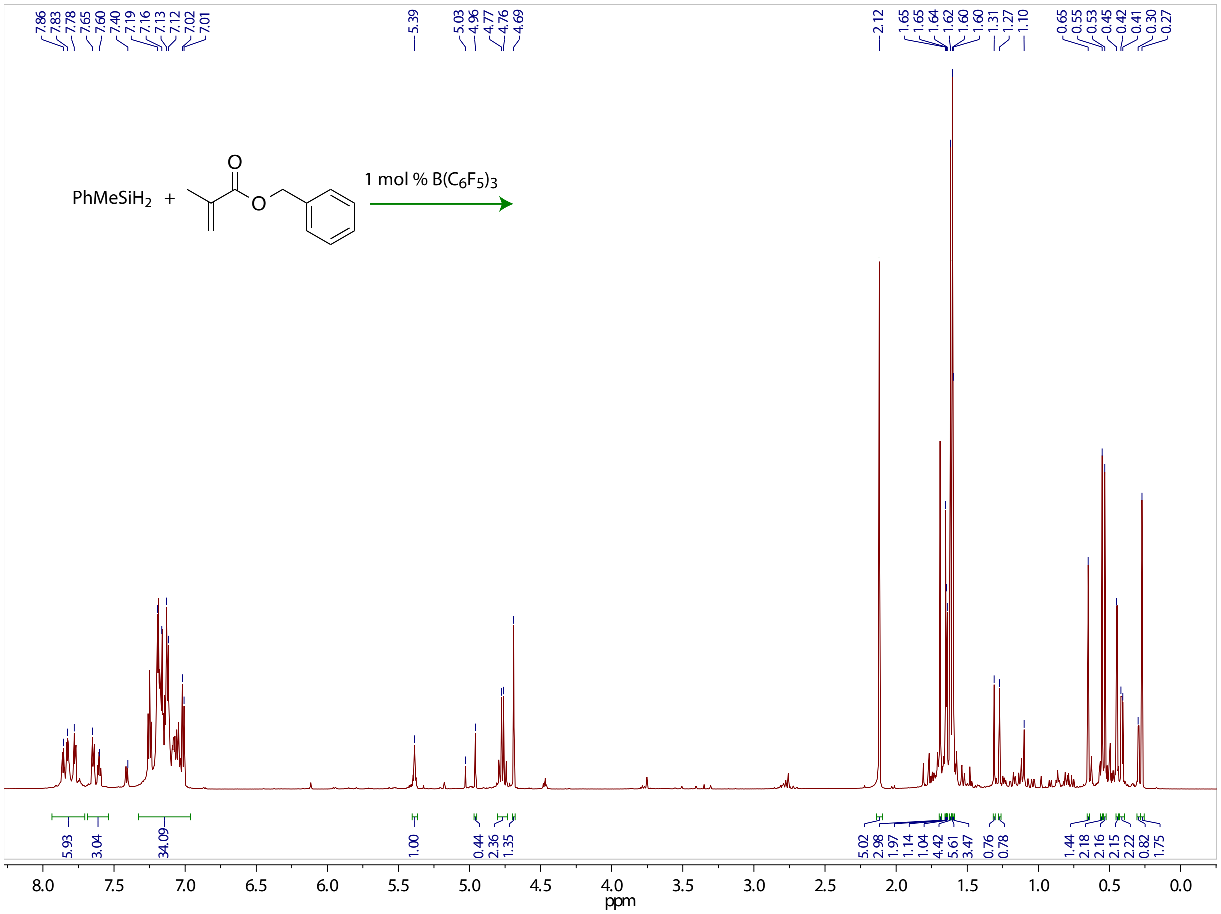


**Figure S20.** $^{1}H$ NMR spectrum of the reaction of $PhMeSiH_2$ and benzyl methacrylate, catalyzed by 1 mol % $B(C_6F_5)_3$. The silyl ketene acetyl is formed as one of a few products.

**3,4-dihydro-6-(phenylmethylsiloxy)-2*H*-pyran.** Crystallized $To^{M}MgHB(C_6F_5)_3$ (0.030 g, 0.033 mmol), 5,6-dihydro-2*H*-pyran-2-one (0.032 g, 0.33 mmol), and $PhMeSiH_2$ (0.042 g, 0.33 mmol) were stirred in benzene for 5 h at room temperature. Evaporation of the volatile materials provided a yellow gel. The product was extracted with pentane, and the extracts were evaporated under reduced pressure to afford a pale yellow liquid (0.059 g, 0.26 mmol, 80.3%). $^{1}H$ NMR (600 MHz, benzene-$d_6$): δ 0.46 (d, 3 H, $^{3}J_{HH}$ = 3.0 Hz, SiMe), 1.32 (pent, 2 H, $^{3}J_{HH}$ = 4.8 Hz, $OCH_2CH_2CH_2CH$=C–OSiHMePh) [CH₂ italic position], 1.80 (m, 2 H, $OCH_2CH_2CH_2CH$=C–OSiHMePh), 3.68 (t, 2 H, $^{3}J_{HH}$ = 4.8 Hz, $OCH_2CH_2CH_2CH$=C–OSiHMePh), 4.10 (t, 1 H, $^{3}J_{HH}$ = 3.6 Hz, $OCH_2CH_2CH_2CH$=C–OSiHMePh), 5.46 (q, 1 H, $^{3}J_{HH}$ = 3.0 Hz, SiH), 7.18 (m, 3 H, $C_6H_5$), 7.66

(m, 2 H, $C_6H_5$). $^{13}C\{^1H\}$ NMR (150 MHz, benzene-$d_6$): δ −1.91 (SiMe), 19.64 ($OCH_2\mathit{C}H_2CH_2CH{=}C$–OSiHMePh), 22.24 ($OCH_2CH_2\mathit{C}H_2CH{=}C$–OSiHMePh), 67.69 ($O\mathit{C}H_2CH_2CH_2CH{=}C$–OSiHMePh), 74.57 ($OCH_2CH_2CH_2\mathit{C}H{=}C$–OSiHMePh), 127.85 ($C_6H_5$), 130.08 ($C_6H_5$), 133.89 ($C_6H_5$), 138.21 (*ipso*-$C_6H_5$), 155.62 ($OCH_2CH_2CH_2CH{=}\mathit{C}$–OSiHMePh). $^{29}Si$ (119 MHz, benzene-$d_6$) δ −5.1 (d, $^1J_{SiH}$ = 218 Hz). IR (KBr, $cm^{-1}$): *ν* 3070 (w), 3050 (w), 2957 (s), 2926 (s), 2854 (m), 2131 (s), 1738 (s), 1637 (w), 1592 (m), 1460 (m), 1429 (s), 1386 (w), 1255 (m), 1121 (s), 1079 (s), 879 (s), 846 (s), 796 (w), 725 (s), 700 (s). Anal. Calcd. for $C_{12}H_{16}O_2Si$: C, 65.41; H, 7.32. Found C, 65.73; H, 6.98.

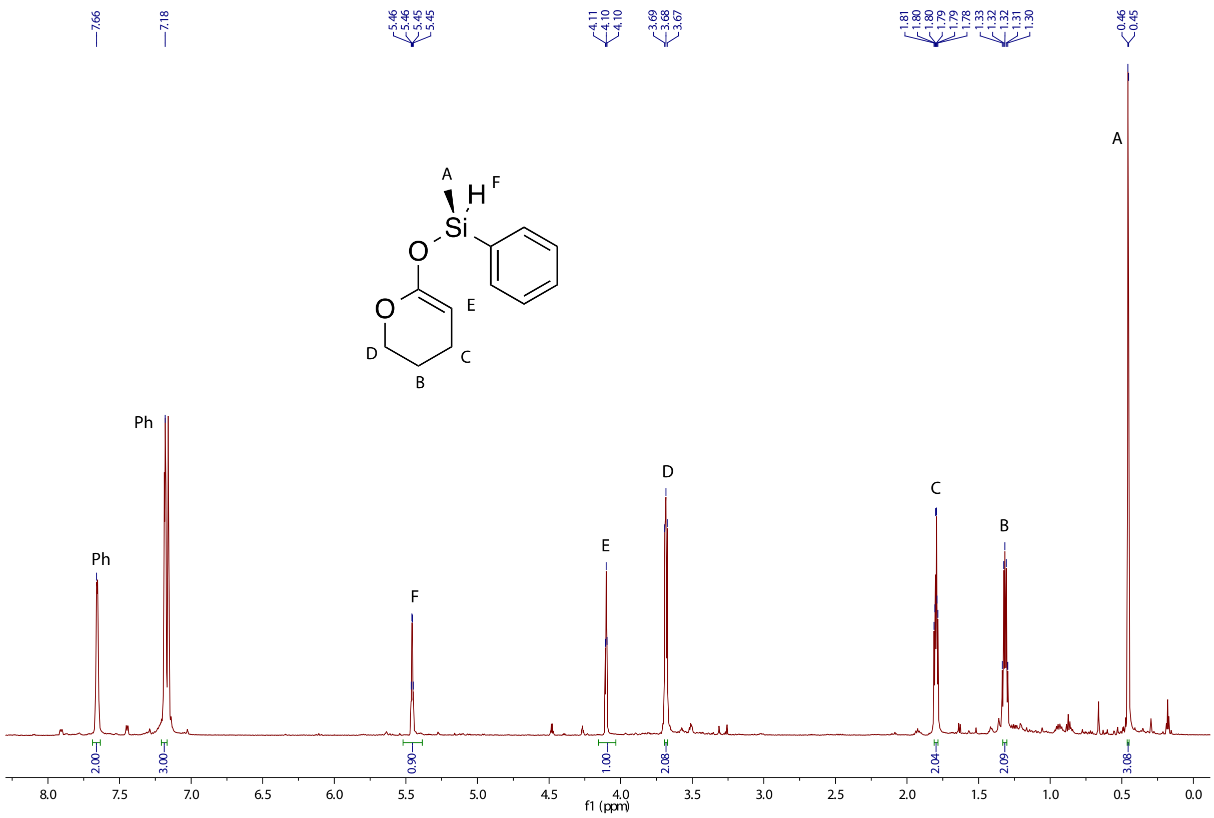


**Figure S21.** $^1H$ NMR spectrum (600 MHz, benzene-$d_6$) of 3,4-dihydro-6-(phenylmethylsiloxy)-2*H*-pyran.

**3,4-dihydro-6-(benzyldimethylsiloxy)-2*H*-pyran.** Crystallized $To^{M}MgHB(C_6F_5)_3$ (0.015 g, 0.015 mmol), 5,6-dihydro-2*H*-pyran-2-one (0.160 g, 1.65 mmol), and $BnMe_2SiH$ (0.245 g, 1.65 mmol) were stirred in benzene for 30 min. at 35 °C. Benzene was removed under reduced pressure, leaving behind a colorless gel. The product was extracted with pentane, and the extracts were evaporated under reduced pressure to afford a colorless liquid (0.335 g, 1.35 mmol, 82.9%). $^1H$ NMR (600 MHz, benzene-$d_6$): δ 0.18 (s, 6 H, $SiMe_2$), 1.39 (pent, 2 H, $^3J_{HH}$ = 4.8 Hz, $OCH_2CH_2CH_2CH{=}C–OSiMe_2Bn$ with $CH_2$ (2nd) italic: $OCH_2C\mathit{H_2}CH_2CH{=}C–OSiMe_2Bn$), 1.86 (m, 2 H, $OCH_2CH_2C\mathit{H_2}CH{=}C–OSiMe_2Bn$), 2.27 (s, 2 H, $SiC\mathit{H_2}Ph$), 3.72 (t, 2 H, $^3J_{HH}$ = 4.8 Hz, $OC\mathit{H_2}CH_2CH_2CH{=}C–OSiMe_2Bn$), 4.02 (t, 1 H, $^3J_{HH}$ = 3.6 Hz, $OCH_2CH_2CH_2C\mathit{H}{=}C–OSiMe_2Bn$), 7.01 (t, 1 H, $^3J_{HH}$ = 7.2 Hz, $C_6H_5$), 7.06 (d, 2 H, $^3J_{HH}$ = 7.2 Hz, $C_6H_5$), 7.14 (t, 2 H, $^3J_{HH}$ = 7.2 Hz, $C_6H_5$). $^{13}C\{^1H\}$ NMR (150 MHz, benzene-$d_6$): δ −1.31 ($SiMe_2$), 20.57 ($OCH_2\mathit{C}H_2CH_2CH{=}C–OSiMe_2Bn$), 23.04 ($OCH_2CH_2\mathit{C}H_2CH{=}C–OSiMe_2Bn$), 27.43 ($Ph\mathit{C}H_2Si$), 67.53 ($O\mathit{C}H_2CH_2CH_2\mathit{C}H{=}C–OSiMe_2Bn$), 74.67 ($OCH_2CH_2CH_2\mathit{C}H{=}C–OSiMe_2Bn$), 125.10 ($C_6H_5$), 128.97 ($C_6H_5$), 129.23 ($C_6H_5$), 139.25 (*ipso*-$C_6H_5$), 155.50 ($OCH_2CH_2CH_2CH{=}\mathit{C}–OSiMe_2Bn$). $^{29}Si$ (119 MHz, benzene-$d_6$) δ 15.6. IR (KBr, $cm^{-1}$): *v* 3081 (w), 3059 (w), 3025(w), 2957 (s), 1733 (s), 1600 (w), 1493 (m), 1452 (m), 1403 (m), 1253 (s), 1208 (s), 1157 (s), 1077 (s), 1057 (s), 907 (m), 838 (s), 762 (s), 700 (s), 619 (w). Anal. Calcd. for $C_{14}H_{20}O_2Si$: C, 67.70; H, 8.12. Found C, 67.77; H, 8.15.

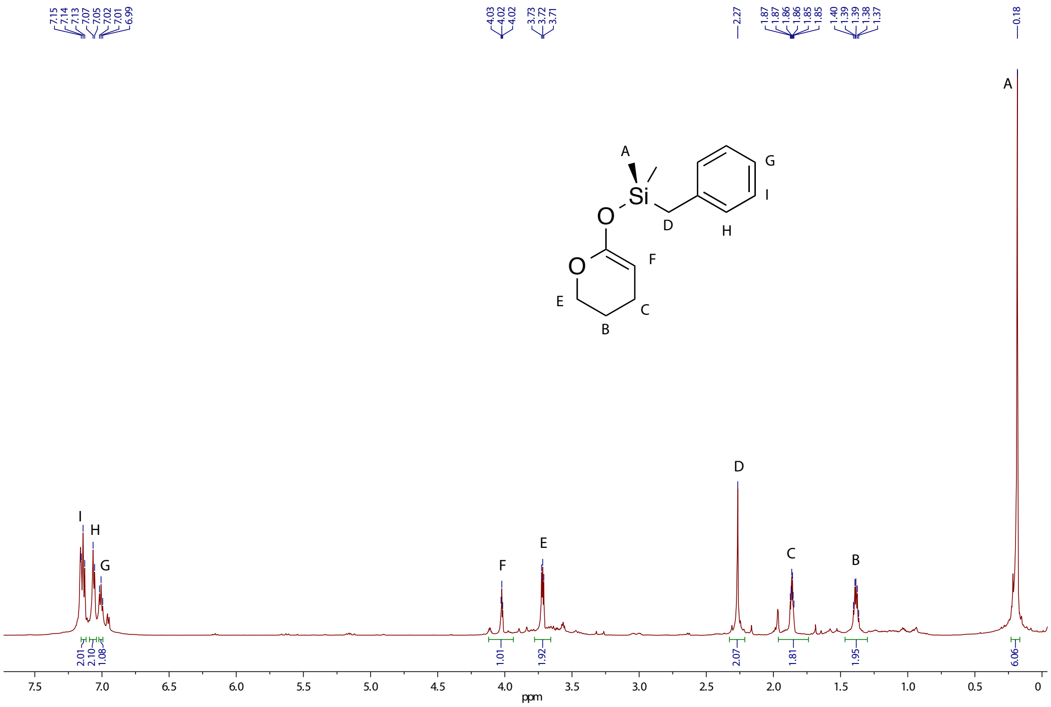


**Figure S22.** $^1$H NMR spectrum (600 MHz, benzene-$d_6$) of 3,4-dihydro-6-(benzyldimethylsiloxy)-2*H*-pyran.

**PhMeSi{O(MeO)C=CMe$_2$}$_2$.**[4] Crystallized To$^M$MgHB($C_6F_5$)$_3$ (0.05 g, 0.054 mmol), methyl methacrylate (0.226 g, 2.18 mmol), and PhMeSiH$_2$ (0.150 g, 1.09 mmol) were stirred in benzene for 12 h at 80 °C. The volatile materials were evaporated under reduced pressure to give a colorless gel. The product was extracted with pentane, and the extracts were evaporated under reduced pressure. Kugelrohr distillation under dynamic vacuum at 180 °C provided a colorless liquid (0.113 g, 0.351 mmol, 32.2%). $^1$H NMR (600 MHz, benzene-$d_6$): δ 0.63 (s, 3 H, SiMe), 1.67 (s, 6 H, C=C*Me*$_2$), 1.69 (s, 6 H, C=C*Me*$_2$), 3.35 (s, 6 H, OMe), 7.19 (m, 3 H, $C_6H_5$), 7.83 (m, 2 H, $C_6H_5$). $^{13}$C{$^1$H} NMR (150 MHz, benzene-$d_6$): δ −3.15 (SiMe), 16.83 (C=C*Me*$_2$), 17.45 (C=C*Me*$_2$), 57.59 (OMe), 92.19 (C=*C*Me$_2$), 128.46 ($C_6H_5$), 131.04 ($C_6H_5$), 134.53 ($C_6H_5$), 134.72 (*ipso*-$C_6H_5$), 150.00 (*C*=CMe$_2$). $^{29}$Si (119 MHz, benzene-$d_6$) δ –20.2. IR (KBr, cm$^{-1}$): *v* 2966 (m), 2925 (s), 2859 (m), 1709 (s), 1593 (w), 1457 (m), 1384 (w), 1261 (s), 1205 (m), 1161

(s), 1125 (m), 1095 (w), 1027 (m), 949 (m), 873 (m), 855 (m), 794 (m), 737 (w), 700 (w). Anal. Calcd. for $C_{17}H_{26}O_4Si$: C, 63.32; H, 8.13. Found C, 62.85; H, 8.43.

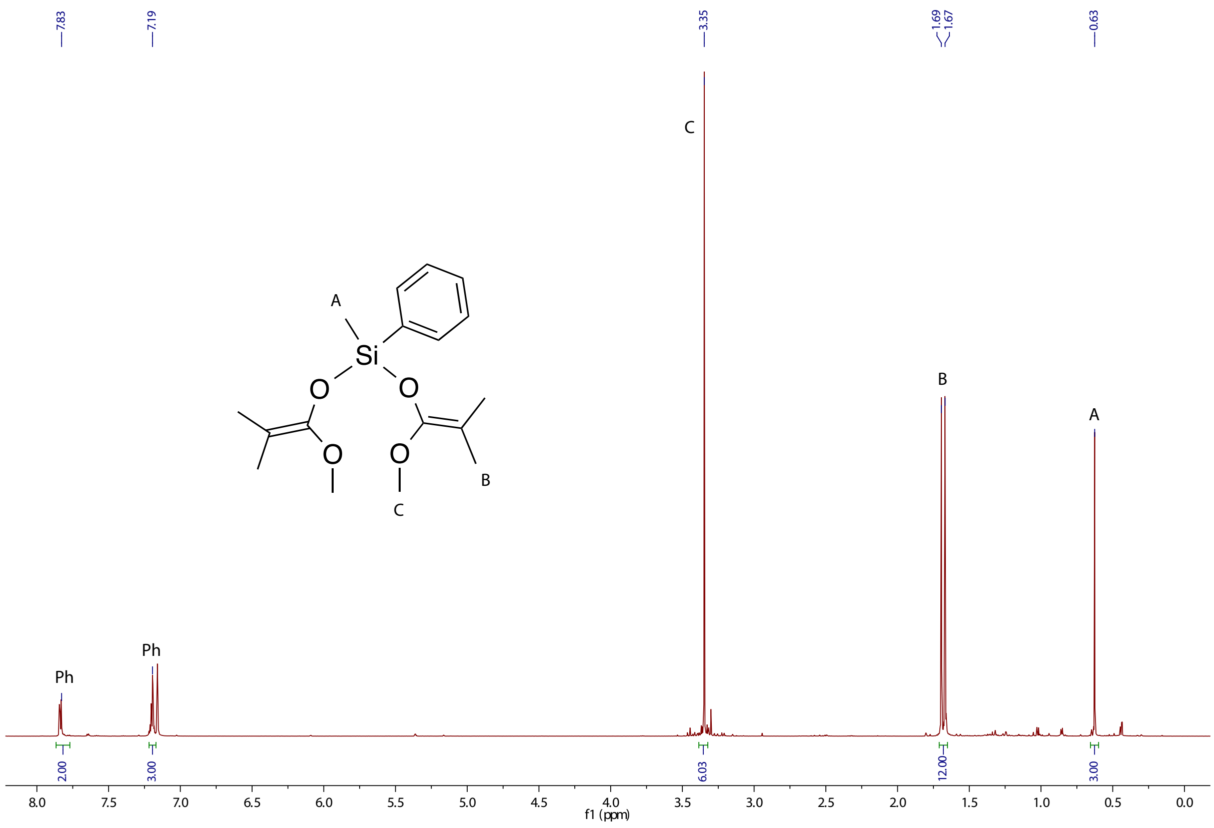


**Figure S23.** $^1$H NMR spectrum (600 MHz, benzene-$d_6$) of PhMeSi{O(MeO)C=CMe$_2$}$_2$.

**References.**


[1] Dunne, J. F.; Fulton, D. B.; Ellern, A.; Sadow, A. D. *J. Am. Chem. Soc.* **2010**, *132*, 17680-17683.

[2] Wang, C.; Erker, G.; Kehr, G.; Wedeking, K.; Fröhlich, R. *Organometallics* **2005**, *24*, 4760-4773.

[3] Sakurai, H.; Shoji, M.; Yajima, M.; Hosomk, A. *Synthesis*, **1984**, 598-600.

[4] Kita, Y.; Yasuda, H.; Sugiyama, Y.; Fukata, F.; Haruta, J.-I.; Tamura, Y. *Tetrahedron Lett.* **1983**, *24*, 1273-1276.
